# Cold Acclimation and Deacclimation of Winter Oilseed Rape, with Special Attention Being Paid to the Role of Brassinosteroids

**DOI:** 10.3390/ijms25116010

**Published:** 2024-05-30

**Authors:** Julia Stachurska, Iwona Sadura, Barbara Jurczyk, Elżbieta Rudolphi-Szydło, Barbara Dyba, Ewa Pociecha, Agnieszka Ostrowska, Magdalena Rys, Miroslav Kvasnica, Jana Oklestkova, Anna Janeczko

**Affiliations:** 1 The Franciszek Górski Institute of Plant Physiology, Polish Academy of Sciences, Niezapominajek 21, 30-239 Krakow, Poland; i.sadura@ifr-pan.edu.pl (I.S.); a.ostrowska@ifr-pan.edu.pl (A.O.); m.rys@ifr-pan.edu.pl (M.R.); 2 Department of Plant Breeding, Physiology and Seed Science, Faculty of Agriculture and Economics, University of Agriculture in Kraków, Podłużna 3, 30-239 Krakow, Poland; barbara.jurczyk@urk.edu.com (B.J.); ewa.pociecha@urk.edu.pl (E.P.); 3 Institute of Biology and Earth Sciences, University of the National Education Commission, Podchorążych 2, 30-084 Krakow, Poland; elzbieta.rudolphi-szydlo@up.krakow.pl (E.R.-S.); barbara.dyba@up.krakow.pl (B.D.); 4 Laboratory of Growth Regulators, Faculty of Science and Institute of Experimental Botany of the Czech Academy of Sciences, Palacký University, Šlechtitelu 27, CZ-78371 Olomouc, Czech Republic; kvasnica@ueb.cas.cz (M.K.); jana.oklestkova@upol.cz (J.O.)

**Keywords:** leaf reflectance, BRI1, *COR*, *SERK*, chlorophyll *a* fluorescence, brassinosteroid analogues, 24-epibrassinolide, 28-homocastasterone, CO_2_ assimilation, frost tolerance

## Abstract

Winter plants acclimate to frost mainly during the autumn months, through the process of cold acclimation. Global climate change is causing changes in weather patterns such as the occurrence of warmer periods during late autumn or in winter. An increase in temperature after cold acclimation can decrease frost tolerance, which is particularly dangerous for winter crops. The aim of this study was to investigate the role of brassinosteroids (BRs) and BR analogues as protective agents against the negative results of deacclimation. Plants were cold-acclimated (3 weeks, 4 °C) and deacclimated (1 week, 16/9 °C d/n). Deacclimation generally reversed the cold-induced changes in the level of the putative brassinosteroid receptor protein (BRI1), the expression of BR-induced *COR*, and the expression of *SERK1*, which is involved in BR signal transduction. The deacclimation-induced decrease in frost tolerance in oilseed rape could to some extent be limited by applying steroid regulators. The deacclimation in plants could be detected using non-invasive measurements such as leaf reflectance, chlorophyll a fluorescence, and gas exchange monitoring.

## 1. Introduction

Oilseed rape (*Brassica napus* ssp. *oleifera* L.) is one of the most important oil crops cultivated in the world—it is a major source of vegetable oil for food and various industries. The plants from winter cultivars produce higher yields, and that is why, e.g., in Poland, they are cultivated more often, but this exposes them to growth in conditions of low temperatures (including frost) during winter. However, the winter species have evolved mechanisms of so-called cold acclimation (cold hardening), which enables them to survive temperatures below 0 °C. The biochemical and physiological processes that accompany cold acclimation are generally well known [1,2]. The more important changes include (1) changes in the membrane lipid composition in the direction of formation of more fluidic cell membranes [3,4], (2) a decrease in the leaf water content along with an increase in the soluble sugar content and the contents of other osmoprotectants [5], (3) an increase in the concentrations of stress hormones such as ABA [6], and (4) an increase in the production of the protective proteins heat shock proteins (HSPs) [7] or cold-responsive proteins (CORs) [8].

Various climate models project that there will be an increase in average global temperatures by at least 2 °C by the end of the 21st century [9]. Global warming in Poland is visible, among other factors, by a rapid increase in the number of days with temperatures higher than 30 °C per year. In the years 1961–90, it was 3.5 days, but between 2011 and 2020 it was already 10.4 days [10]. Global warming is also connected with fluctuations in temperatures in autumn, for example, in regions of the eastern EU. Normally, a systematic drop in temperature to cold (more than zero) is a natural condition for cold acclimation. A period of 3–6 weeks of cold acclimation with a temperature of about 4 °C is usually needed for winter oilseed rape. After such a period, plants become much more frost-tolerant than before the cold acclimation and are able to survive the winter frost [11]. Because of the aforementioned climate changes, the cold acclimation process could be disturbed by sudden episodes of higher temperatures, such as 16 °C or more, e.g., during autumn and early winter, which induces the deacclimation (dehardening) process. This phenomenon decreases the frost tolerance of plants [11,12]. Deacclimation depends on the duration (number of days with higher temperatures) or the range of the higher temperatures [12]. For example, the deacclimation rate is faster at 20 °C than at 12 °C [12]. Longer periods of deacclimation could even lead plants to the resumption of growth and development.

As was mentioned earlier, although the metabolic background of cold acclimation is quite well known, more in-depth studies that are devoted to the mechanisms of the reactionof plants to deacclimation are relatively new and not numerous. To date, research has been conducted on the model plant *Arabidopsis thaliana* (L.) (among others [13,14]) and crop plants such as oilseed rape [12,15], barley [16], and wheat [17]. Regarding oilseed rape, our earlier studies have shown that deacclimation increases the efficiency of the light and dark reactions of photosynthesis, while on the metabolic level it causes changes in hormonal homeostasis, soluble sugars, or the accumulation of HSPs, although there is a revertive effect compared to the effect that is generated by cold [11,15,18]. For example, as a result of deacclimation, there was a decrease in the concentrations of the stress hormones (mainly ABA) and an increase in the growth-promoting hormones [18]. Such a shift in the hormonal balance as a result of deacclimation could be one of the possible causes of the decreased frost tolerance in deacclimated oilseed rape plants. This could happened especially when it is accompanied by a decrease in the concentration of soluble sugars, which have osmoprotective properties, and a decrease in the accumulation of the protective proteins [15].

The current work is a continuation of studies on the metabolic background of deacclimation in this economically important species (oilseed rape). In this study, however, we focused more on the possible role of brassinosteroids in the process of the cold acclimation and deacclimation of oilseed rape, along with the possibilities of using these regulators to counteract the negative effects of frost. It is worth emphasising that although the steroid hormones brassinosteroids (BRs) were first discovered in oilseed rape’s pollen [19], their physiological role in this species is still rather poorly explained. The group of brassinosteroids includes 81 compounds. Brassinosteroids are characterised by various numbers of carbon (C) atoms in a molecule. There are three main groups of brassinosteroids: C27, C28, and C29 [20,21]. For example, brassinosteroids include brassinolide, castasterone, 24-epibrassinolide, and 28-homocastasterone. Synthetic analogues of BRs, the physiological activity of which is still being tested [22], are also available. Generally, the role of brassinosteroids in plants is important for regulating their growth and development, while BRs also have a protective effect in plants that are growing under stressful conditions such as drought [23], salinity [24], heavy metal stress [25,26], or low temperatures [27,28]. Regarding low-temperature stress, a limited number of studies have been devoted to the role of BRs as players that prepare the metabolism of plants for subzero temperatures during the cold acclimation/hardening process [20]. The effect of BRs on frost tolerance in cold-acclimated plants has been studied in the monocot plants of a group of cereals [28,29], as well as in dicot plants (*A. thaliana*, basal frost tolerance) [30]. As for oilseed rape, it is known that cold acclimation reduces the accumulation of the *BRI1* transcript (encoding the BR receptor protein) [11]. After deacclimation, in more frost-susceptible cultivars, the level of the *BRI1* transcript increases once again, but in more frost-tolerant cultivars, the level of *BRI1* remains low, similar to what is observed in the cold-acclimated plants [11]. Interestingly, cold acclimation increased the concentrations of some of the brassinosteroids in oilseed rape, while deacclimation most often decreased their levels [11].

The current article presents the results of a cycle of experiments that were conducted on non-acclimated (NA), cold-acclimated (CA), and deacclimated (DA) oilseed rape, in which the aims were as follows:

1. To examine a few brassinosteroids (24-epibrassinolide (EBR) and 28-homocastasterone (HCS)) and brassinosteroid analogues (triolon (TR) and MK-266 (MK)) as potential agents that exhibit a protective activity against frost in NA plants (EBR), CA plants (EBR), and DA plants (EBR, HCS, MK, TR).
2. To describe the impact of steroids on the physicochemical properties of membranes, including describing the interaction of two BR analogues with the defined model lipid monolayers (mimicking cell membranes) in order to assess their influence on the fluidity of the lipid monolayers, which are important from the point of view of frost tolerance.
3. To describe the activity of the abovementioned steroids in regulating photosynthesis and the leaf spectral properties during the cold acclimation and deacclimation of oilseed rape.
4. To characterise any cold-acclimation-induced and deacclimation-induced changes in (a) the accumulation of the protein BRI1 (brassinosteroid membrane receptor), (b) the expression of the genes encoding the proteins that participate in BR signalling (*SERK1* and *SERK2*), and (c) the expression of the BR-dependent genes (*COR*).
5. To verify the possibility of using non-invasive measurement techniques (i.e., leaf reflectance) for the detection of deacclimation in plants.

## 2. Results

### 2.1. The Influence of BR and BR Analogues on Frost Tolerance

In the current research, both of the non-acclimated cultivars, Pantheon and President, suffered only minor injuries to their leaves, and on the regrowth scale they reached values close to the maximal seven points (control abs) (Table 1, Figure S1). In both cultivars, application of water with DMSO (control) statistically significantly decreased plant survival by roughly one point. The application of EBR reduced this effect, but only in the cultivar President. There were, however, no statistically significant differences between the plants of the control abs and the EBR-sprayed plants.

The CA plants were able to survive −13 °C with a score that ranged between 3.00 and 4.69 points (Table 1). In both cultivars, spraying the plants with water containing DMSO (control) was not beneficial and lowered frost tolerance (by about 0.7 points compared to the control abs). Interestingly, EBR reduced this effect. However, when the untreated plants (control abs) and the EBR-sprayed plants were compared, EBR improved the frost tolerance in only one cultivar (President). The EBR-treated plants of this cultivar had a score more than 0.8 points higher than the control abs (Table 1, Figure S1).

The oilseed rape plants that had been deacclimated and then exposed to frost of −6 °C were able to survive with a score between 2 and 5 points (Table 1). The DA plants that had been treated with EBR had higher scores compared to both the control abs and control. No cultivar dependency was observed (Figure S1). However, the effect of EBR was, weaker when the plants were exposed to −9 °C, and it disappeared at −12 °C (regrowth between 1 and 2 points) (Table 1, Figure S1).

Other regulators were also tested on the DA plants: the brassinosteroid HCS, detected earlier in the leaves of oilseed rape [11]; two BR analogues (MK, TR); and the commercial plant growth regulator Asahi (AS). The solvent of steroids (DMSO in the DA control) had some effect compared to the untreated plants (DA control abs), particularly after the frost tests at −6 °C (Pantheon, weakened regrowth) and −9 °C (President, even better regrowth).

Plants of the cultivar Pantheon responded well to the application of the tested regulators in the case of a milder frost. Improved plant regrowth was observed for all of them after the frost test at −6 °C (compared to the control with DMSO), and for MK, TR, and AS compared to the control abs (Table 1). After the frost tests at −9 °C, HCS, MK, and AS were effective. Frost at −12 °C caused severe injuries; however, similar to the case of EBR, the other regulators were no longer effective. Statistically significant negative effects of HCS and MK were even observed.

As for the cultivar President, improved plant regrowth after the frost test at −6 °C was observed for HCS, TR, and AS (compared to the control abs), but only for HCS in comparison to the control with DMSO (Table 1). After the frost tests at −9 °C, compared to the untreated plants, all of the steroid regulators were effective, but due to the relatively good regrowth of the plants of the control with DMSO, there were no statistically significant differences between this control and the plants that had been sprayed. Frost at −12 °C caused severe injuries, and the regulators were no longer effective. Statistically significant negative effects of MK and AS were even observed in comparison to the control abs.

### 2.2. The Effects of BRs and BR Analogues on Membrane Permeability

After 3 h from the moment of freezing, the electrolyte leakage in the NA control abs leaves reached a value of 53.75 µS (Table 2). After cold acclimation, the electrolyte leakage in the control abs leaves reached a value of 2.82 µS, while after deacclimation it was at a similar level as in the CA leaves (2.25 µS). Application of DMSO to the NA leaves (control) resulted in a similar value to that in the control abs (57.82 µS). Moreover, in the CA and DA leaves, DMSO did not significantly affect the electrolyte leakage when compared with the control abs. Application of EBR and HCS to the NA and DA plants did not change the electrolyte leakage values compared to the control leaves, but in the CA plants EBR and HCS significantly increased its values. Another regulator, MK, significantly increased the electrolyte leakage in the leaves of the NA, CA, and DA plants compared with control and control abs. TR did not affect the electrolyte leakage in the NA and CA plants, while in the DA plants its application caused the highest electrolyte leakage.

Electrolyte leakage 24 h after freezing in the NA control abs leaves reached a value of 128.24 µS (Table 2). In the control abs plants, after cold acclimation, it was lower than in the NA plants (only 5.54 µS). After deacclimation, the electrolyte leakage in the control abs leaves also was lower than in the leaves of the NA plants. DMSO slightly increased the electrolyte leakage in the NA and CA plants, while it did not affect the DA plants when compared to the control abs. EBR and HCS did not increase the electrolyte leakage in the leaves of the NA plants. In the leaves of the CA plants, those regulators caused a significantly higher electrolyte leakage than in the control and control abs. EBR and HCS did not affect the DA plants. The BR analogue MK significantly increased the electrolyte leakage in the leaves of the NA, CA, and DA plants compared to the control abs and control. TR increased the electrolyte leakage in DA plants.

### 2.3. Interaction of BR Analogues (MK, TR) with the Model Membranes

**Figure 1:**
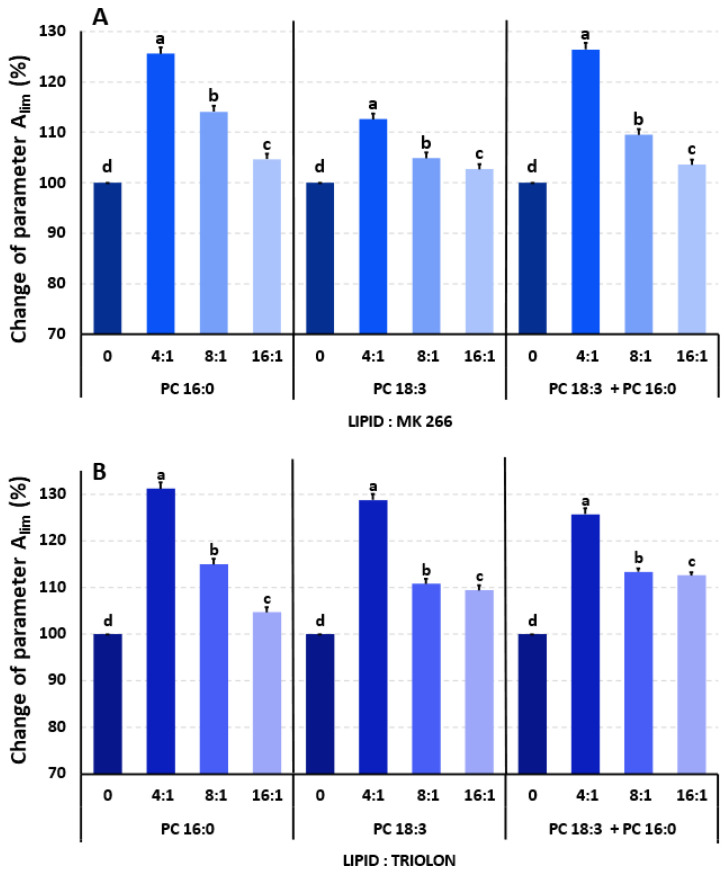
The influence of BR analogues on the limiting area per molecule (parameter A_lim_) of the model monolayers. The control system (marked 0), considered to be 100%, was represented by the lipid isotherm on the subphase without the addition of hormones. (**A**) Mixture of lipid and MK-266; (**B**) Mixture of lipid and triolon. Percentage values were calculated based on the original data presented in Table S1. Statistically significant differences between systems (Duncan’s test, *p* ≤ 0.05) are indicated by different letters; statistical analysis was conducted separately for each group: PC 16:0, PC 18:3, and PC 18:3 + PC 16:0.

The exemplary isotherms of surface pressure (π) as a function of surface area per lipid molecule in a monolayer for PC 18:3, PC 16:0, and their mixtures are presented in Figures S2 and S3. Based on these isotherms, the physicochemical parameters such as A_lim_, π_coll_, and C_s_^−1^ were calculated and are presented in Table S1. The percentage changes in the A_lim_ parameter, which indicates the surface area that is occupied by the lipids in the monolayer, are presented in Figure 1.

Adding the analogues MK-266 and triolon to the single- and double-compound lipid mixtures, consisting of saturated and unsaturated lipids, resulted in an increase in the A_lim_ parameter (Figure 1A,B, Table S1). Higher hormone concentrations were associated with a greater increase in the A_lim_ value, which ranged from approximately 2% to around 31% and was concentration-dependent. The most significant changes in these experimental systems were observed for the 4:1 mixture (M:M).

In the case of the second determined parameter, π_coll_, the changes that were observed in the studied systems were slight but were statistically significant compared to the control (system without the addition of hormones). Changes in the pressure at which the monolayer collapsed ranged from approximately ±0.5 to 3 mN/m (Table S1).

For a more detailed analysis of changes occurring under the influence of hormone addition, stability curves of the compression modulus (C_s_^−1^) were plotted against surface pressure, as depicted in Figure S3 (and, as numerical results, in Table S1).

### 2.4. Accumulation of the Brassinosteroid Receptor Protein (BRI1)

**Figure 2:**
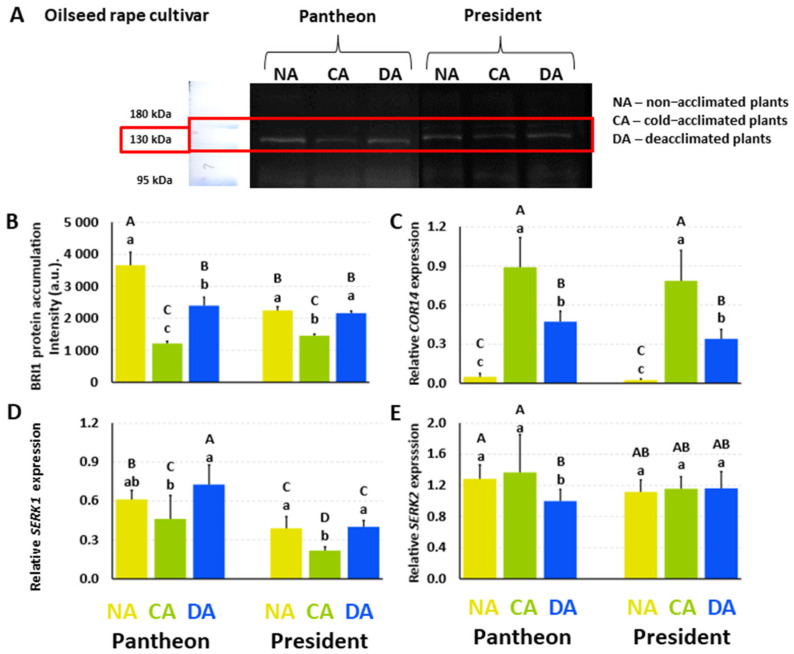
The accumulation of the putative BRI1 protein and relative gene expression (*COR14*, *SERK1*, *SERK2*) in the leaves of the non-acclimated (NA), cold-acclimated (CA), and deacclimated (DA) oilseed rape plant cultivars Pantheon and President. (**A**) The representative original membrane, where the visualised bands correspond to the level of putative BRI1 protein; 15 µg of protein was loaded onto the gel. (**B**) The accumulation of the putative BRI1 protein. (**C**) Relative expression of *COR14*. (**D**) Relative expression of *SERK1*. (**E**) Relative expression of *SERK2*. The transcript levels were calculated relative to actin (endogenous reference gene). Mean values ± SE that are indicated by the same letters did not differ according to Duncan’s test (*p* < 0.05). Lowercase letters—comparisons between the NA, CA, and DA plants within each cultivar. Capital letters—comparisons between the NA, CA, and DA plants of both cultivars.

In the NA Pantheon plants, the accumulation of the putative BRI1 protein was at a level of 3657 (a.u.) (Figure 2A,B). After cold acclimation, the accumulation of this protein decreased significantly by 67%, while after deacclimation the putative BRI1 accumulation increased significantly, by almost 100%; however, it did not reach the level that was observed in the NA plants. In the NA President plants, the accumulation of putative BRI1 reached a level of 2242 (a.u.) (Figure 2B). After cold acclimation, the amount of this protein decreased by 35%. However, after deacclimation, the accumulation of putative BRI1 increased significantly and reached a value that was similar to what was detected in the NA plants. In addition to the cultivars Pantheon and President, BRI1 was also analysed for two other winter cultivars (Bojan, Rokas) and one spring cultivar (Feliks). A similar model of changes in the putative BRI1 accumulation to those that were observed in the NA, CA, and DA plants of Pantheon and President was confirmed in these three additional cultivars (Figure S4).

### 2.5. Expression of the Genes Encoding the Proteins That Participate in BR Signalling (SERK1 and SERK2) and the BR-Dependent Gene (COR14)

The relative expression of the *COR14* gene in the NA Pantheon plants reached a value of 0.0494 (Figure 2C). After cold acclimation, there was a significant increase (18-fold) in the *COR14* expression. In the DA plants, the amount of this transcript decreased by almost 50% compared to the CA plants. A similar tendency was observed in the cv. President—in the NA plants, the amount of the *COR14* transcript reached a value of 0.0236, while after cold acclimation there was a significant increase of about 30-fold. In the DA President plants, the expression of *COR14* decreased (about 50%), but it did not reach the level that was characteristic of NA plants.

In the NA Pantheon plants, the *SERK1* expression reached a value of 0.611 (Figure 2D). After cold acclimation, the amount of this transcript decreased significantly, by 25%. However, after deacclimation, the expression of *SERK1* increased to a level that was similar to that of the NA plants. A similar tendency of changes in the *SERK1* expression was observed in the cultivar President. In the NA President plants, the expression of *SERK1* reached a value of 0.3882. After cold acclimation, the expression significantly decreased by 44%, while after deacclimation it increased once again and reached a level similar to that of the NA plants. The expression of *SERK2* in the NA Pantheon plants reached a value of 1.284 (Figure 2E), and after cold acclimation it did not change significantly. In the DA Pantheon plants, the *SERK2* expression decreased significantly, by 27% compared to the CA plants. In the cultivar President, the expression of *SERK2* that was detected in the NA plants reached a value of 1.114, and it did not change significantly in the CA and DA plants.

### 2.6. The Effects of BRs and BR Analogues on PSII Efficiency

The so-called parameters of yield, or flux ratios (φ_Po_, ψ_o_, and φ_Eo_), were calculated based on data from fluorescence curves.

**Figure 3:**
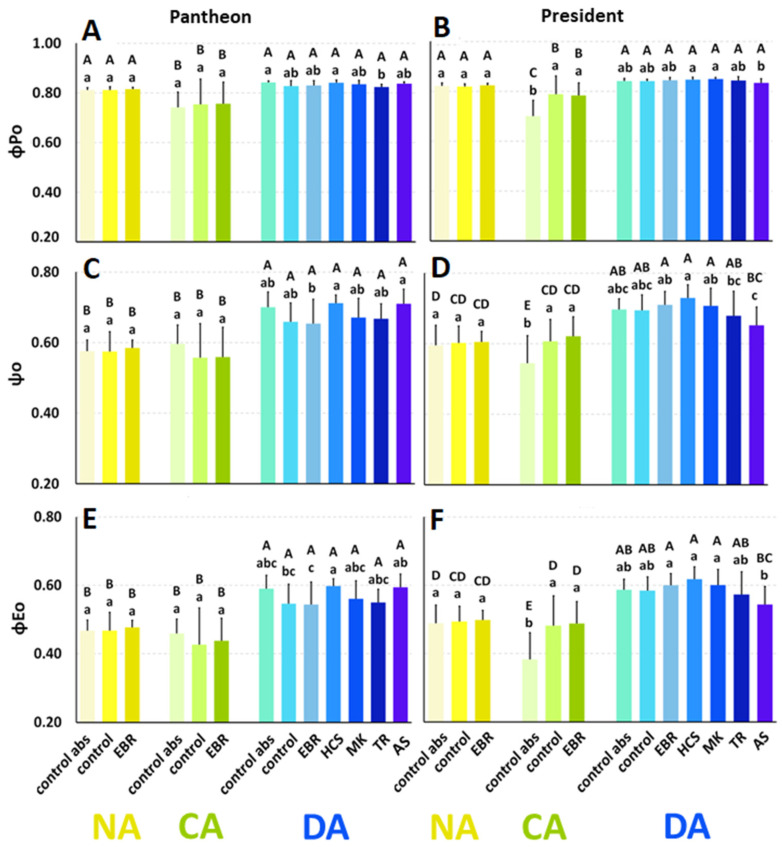
Quantum photosynthetic efficiency described by the chlorophyll *a* fluorescence measurements (yield/flux ratios [31]) of the non-acclimated (NA), cold-acclimated (CA), and deacclimated (DA) winter oilseed rape cultivars Pantheon (**A**,**C**,**E**) and President (**B**,**D**,**F**); φ_Po_—maximum quantum yield of primary photochemistry (at t = 0) (**A**,**B**); ψ_o_—probability (at t = 0) that a trapped exciton moves an electron into the electron transport chain beyond Q_A_^−^ (**C**,**D**); φ_Eo_—quantum yield of electron transport (at t = 0) (**E**,**F**). Control abs—untreated plants; control—plants treated with a water solution of DMSO (a solvent of the tested steroids). The other objects represent plants that had been sprayed with brassinosteroids (EBR—24-epibrassinolide; HCS—28-homocastasterone), brassinosteroid analogues (MK—MK-266; TR—triolon), and the regulator Asahi SL (AS). Mean values indicated by the same letters did not differ significantly according to Duncan’s test (*p* < 0.05). Lowercase letters—comparisons between the treatments within a specific group (NA, CA, and DA plants); capital letters—comparisons between the treatments of the plants in all three groups (NA, CA, and DA plants).

The maximum quantum yield of primary photochemistry (φ_Po_) in the cultivar Pantheon (NA control abs) reached a value of 0.81, and after cold acclimation it significantly decreased by about 9% (Figure 3A). After deacclimation, the φ_Po_ value returned to the level that was characteristic of the NA plants. The application of the working solutions with DMSO (control plants) did not cause any changes in the φ_Po_ value in the NA, CA, and DA plants. The application of the tested regulators did not influence the φ_Po_ values (compared to the control plants).

In the NA President plants (control abs), the value of φ_Po_ reached 0.82 (Figure 3B). After cold acclimation, φ_Po_ significantly decreased by 15%, while after deacclimation φ_Po_ increased and reached a value that was similar to that of the NA plants. The application of DMSO did not affect the φ_Po_ values in the NA and DA plants, but in the CA plants φ_Po_ was increased by about 13% compared to the control abs. No effect of the tested regulators on φ_Po_ was observed.

The probability that a trapped exciton moves an electron into the electron transport chain beyond Q_A_^−^ (ψ_o_) in the NA Pantheon plants (control abs) reached a value of 0.58 (Figure 3C). After cold acclimation, it did not change significantly, but after deacclimation ψ_o_ increased to a value that was 22% higher than in the NA plants. There was no effect of the DMSO treatment (control) on the NA, CA, and DA plants. The application of the regulators did not change ψ_o_.

In the cultivar President, in the NA control abs plants, the ψ_o_ value was 0.59 (Figure 3D). After cold acclimation, it decreased significantly by 8%, while after deacclimation it increased to a value of 0.70, which was 17% higher than in the NA plants. The DMSO application did not affect ψ_o_ in the NA and DA plants, but it significantly increased the ψ_o_ value in the CA plants compared to the control abs plants (by 11%). The application of the regulators did not change ψ_o_ significantly.

The quantum yield of electron transport (φ_Eo_) in the NA Pantheon control abs plants reached a value of 0.47, and it did not change after cold acclimation (Figure 3E). After deacclimation, φ_Eo_ increased by 26%. In comparison to the absolute control, there was no effect of DMSO (control) and the applied regulators on φ_Eo_.

In the NA control abs plants of the cultivar President, the value of φ_Eo_ reached 0.49, and it decreased by 22% after cold acclimation (Figure 3F). After deacclimation, φ_Eo_ increased once again and reached a value 20% higher compared to the NA plants. The DMSO did not affect φ_Eo_ in the NA plants, but in the CA plants it increased φ_Eo_ by 26%. The tested BRs and BR analogues did not induce any significant changes in the values of φ_Eo_.

### 2.7. The Effects of BRs and BR Analogues on Leaf Gas Exchange

**Figure 4:**
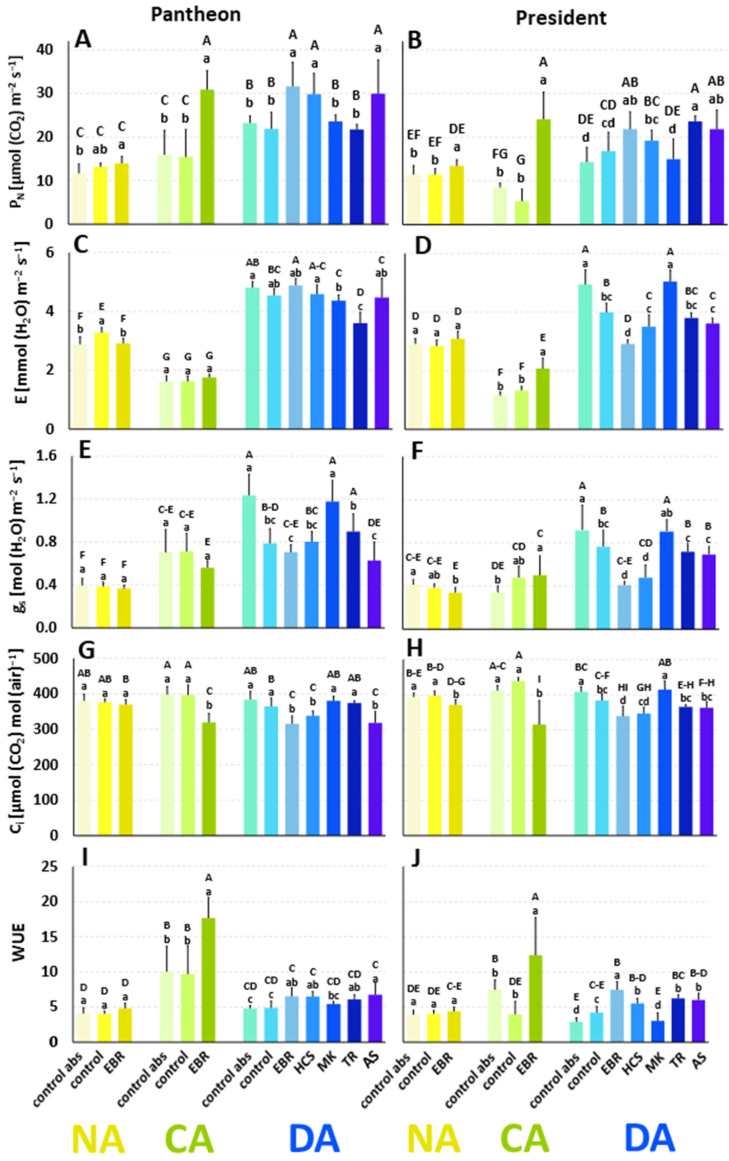
Gas exchange in the non-acclimated (NA), cold-acclimated (CA), and deacclimated (DA) leaves of the oilseed rape cultivars Pantheon (**A**,**C**,**E**,**G**,**I**) and President (**B**,**D**,**F**,**H**,**J**). P_N_—net photosynthesis intensity (**A**,**B**); E—transpiration (**C**,**D**); g_s_—stomatal conductance (**E**,**F**); C_i_—intracellular concentration of CO_2_ (**G**,**H**); WUE—photosynthetic ratio of water use (**I**,**J**). Control abs—untreated plants; control—plants that were treated with a water solution of DMSO (a solvent of the tested steroids). The other objects represent plants that had been sprayed with brassinosteroids (EBR—24-epibrassinolide; HCS—28-homocastasterone), brassinosteroid analogues (MK—MK-266; TR—triolon), and the regulator Asahi SL (AS). Mean values indicated by the same letters did not differ significantly according to Duncan’s test (*p* < 0.05). Lowercase letters—comparisons between the treatments within a specific group (NA, CA, and DA plants); capital letters—comparisons between the treatments of plants of all three groups (NA, CA, and DA plants).

Net photosynthesis intensity (P_N_) is a parameter that provides information about the intensity of the CO_2_ assimilation. In the cultivar Pantheon, in the NA plants (control abs), the value of P_N_ reached 11.67 µmol (CO_2_) m^−2^ s^−1^ (Figure 4A). After cold acclimation, in the control abs plants, P_N_ increased by about 36%, while after deacclimation there was a further increase by about onefold in comparison to the NA plants. The application of DMSO did not affect P_N_ in the NA, CA, or DA plants. The application of EBR significantly increased P_N_ in the NA, CA, and DA plants. The other regulators that were used in the DA plants, HCS and AS, also caused an increase in P_N_, while the application of MK and TR did not affect this parameter.

In the NA President plants (control abs), the value of P_N_ reached 11.41 µmol (CO_2_) m^−2^ s^−1^ (Figure 4B). After cold acclimation, P_N_ decreased by about 25%. After deacclimation, P_N_ increased again and reached values of about 25% higher compared to the NA plants. The application of DMSO did not significantly affect the P_N_ values in the NA, CA, and DA plants. The application of EBR improved P_N_ in the NA, CA, and DA plants. The application of the other regulators, TR and AS, to the DA plants increased P_N_. The application of MK did not affect P_N_ in the DA President plants.

Transpiration intensity (E) in the NA control abs plants of the Pantheon cultivar reached 2.88 mmol (H_2_O) m^−2^ s^−1^, while after cold acclimation it decreased by 44% (Figure 4C). However, after deacclimation, there was generally about a twofold increase in E when compared to the CA plants. The application of DMSO increased E in the NA plants very slightly, but it did not affect the CA and DA plants. The application of EBR decreased E in the NA plants but did not have a significant effect in the CA and DA plants compared to the controls. Plants sprayed with the other regulators, HCS and AS, had E values similar to the controls. MK and TR even caused a decrease in E.

In the cultivar President, the NA plants were characterised by E reaching 2.92 mmol (H_2_O) m^−2^ s^−1^ (Figure 4D). After cold acclimation, E significantly decreased, by about 60%. After deacclimation, in the control abs plants, E increased by about threefold when compared to the CA plants. The application of DMSO did not affect E in the NA and CA plants, but it caused a decrease in E in the DA plants. Although the application of EBR did not affect the NA plants, it caused a significant increase in E in the CA plants. In the DA plants, there was a significant decrease in E after the application of EBR. The application of HCS, TR, and AS caused a decrease in E, while MK caused an increase in E when compared to the control plants.

Stomatal conductance (g_s_) in the control abs of NA Pantheon plants reached a value of 0.39 mol (H_2_O) m^−2^ s^−1^ (Figure 4E). After cold acclimation, g_s_ in the control abs plants increased by 78%, while after deacclimation it further increased by 76% compared to the CA plants. The application of DMSO did not affect g_s_ in the NA and CA plants; however, in the DA plants, it decreased g_s_ significantly, by about 37%, compared to the control abs. The use of EBR did not cause significant changes in g_s_ in the NA, CA, and DA Pantheon plants. Among the other regulators that were used in the DA Pantheon plants, HCS, TR, and AS did not change the g_s_ values, while MK increased g_s_.

The value of g_s_ in the control abs of NA President plants was 0.41 mol (H_2_O) m^−2^ s^−1^ (Figure 4F), and after cold acclimation, unlike the Pantheon plants, it did not change significantly. However, after deacclimation, in the control abs plants, g_s_ increased significantly by about 170% compared to the CA plants. Similar to the Pantheon plants, the application of DMSO did not affect g_s_ in the NA and CA plants, but it decreased g_s_ significantly (by about 17%) in the DA plants. In the NA and CA President plants, the application of EBR did not change the values of g_s_ compared to the control plants. However, in the DA plants, EBR decreased the values of g_s_ to a level that was similar to those in the NA plants and even in the CA plants. The application of HCS, TR, and AS decreased the g_s_ value compared to the control abs, and also partially compared to the plants that were treated with DMSO. The plants that were treated with MK were characterised by a level of g_s_ that was similar to the control abs.

The intracellular concentration of CO_2_ (C_i_) in the plants of the control abs (NA, Pantheon) was 380 µmol (CO_2_) mol (air)^−1^ (Figure 4G). In the CA plants, it remained similar (398 µmol (CO_2_) mol (air)^−1^), and C_i_ did not change significantly after deacclimation. There was no effect of DMSO on the values of C_i_ in the NA, CA, and DA Pantheon plants compared to the control abs. EBR did not affect the values of C_i_ in the NA plants; however, the steroid did decrease C_i_ in the CA and DA plants, similar to HCS and AS. There was no effect of MK and TR on the values of C_i_ in the DA plants compared to both controls.

In the cultivar President, C_i_ did not vary significantly between the NA, CA, and DA plants of the control abs (Figure 4H). The application of DMSO did not affect the NA and CA plants; however, it caused a slight, but significant decrease in C_i_ (6%) in the DA plants. The application of EBR affected the NA, CA, and DA plants; the hormone caused significant decreases in C_i_. The other BR (HCS) caused a decrease in C_i_ compared to the control abs. The BR analogue (TR) that was used in the DA plants did not generally change the C_i_ values. Only MK increased C_i_ compared to the control.

The value of water-use efficiency (WUE) reached 4.09 in the NA control abs plants of the cultivar Pantheon (Figure 4I). After cold acclimation, there were increases in WUE for the control abs and control (DMSO) plants by about 140%, while for the EBR-treated plants the increase was more than 260%. After deacclimation, WUE generally decreased to values that were similar to those of the NA plants. The application of DMSO did not change the WUE values in the NA, CA, and DA plants when compared to the control abs. In the NA plants, EBR did not affect WUE, while in the CA plants it caused an increase in this parameter. After deacclimation, the application of EBR increased the WUE. The application of all of the regulators (except for MK) slightly increased the values of WUE (by an average of 33%) in the DA plants compared to both of the controls.

In the control abs plants of the President cultivar, the WUE value was 3.91 (Figure 4J). Cold acclimation resulted in an increase in the WUE values; in the control abs plants, there was almost a 90% increase. After deacclimation, the WUE in the control abs plants decreased to a level that was even lower than in the NA plants. The application of DMSO did not affect the NA and CA plants, while it resulted in higher WUE in the DA plants when compared to the control abs plants. No effect of EBR was observed in the NA plants. The application of EBR caused an increase in the WUE value in the CA plants, and a similar effect was observed after deacclimation. Higher WUE was also observed in the DA plants that were treated with HCS, TR, and AS.

### 2.8. The Effects of BRs and BR Analogues on the Leaf Spectral Properties

**Figure 5:**
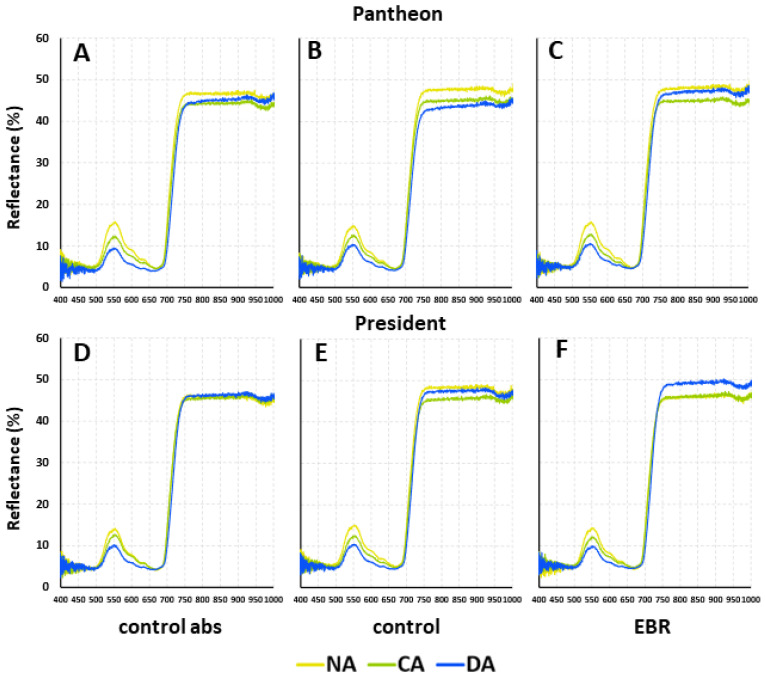
The reflectance curves expressing the reflectance intensity (%) of the leaves of non-acclimated (NA), cold-acclimated (CA), and deacclimated (DA) oilseed rape. (**A**–**C**) Pantheon cultivar; (**D**–**F**) President cultivar. X-axis = wave length [nm]. Control abs—untreated plants; control—plants sprayed with a water solution of DMSO (a solvent of the tested steroids); EBR—plants sprayed with the steroid hormone 24-epibrassinolide. Each curve represents the average of the measurements that were taken on ten leaves (each replicate was one leaf from different plants).

The reflectance intensity of the leaves of the cultivars Pantheon and President increased in the range of 500–550 nm for the NA, CA, and DA plants (Figure 5). Then, it decreased in the range of 550–700 nm, increased sharply in the range of 700–750 nm, and reached a plateau at the range of 750–950 nm for both the control abs and control, as well as in EBR-treated plants. Slight visible differences were observed in the reflectance intensity between the NA, CA, and DA plants, especially in the ranges of 500–650 nm and 750–950 nm.

**Figure 6:**
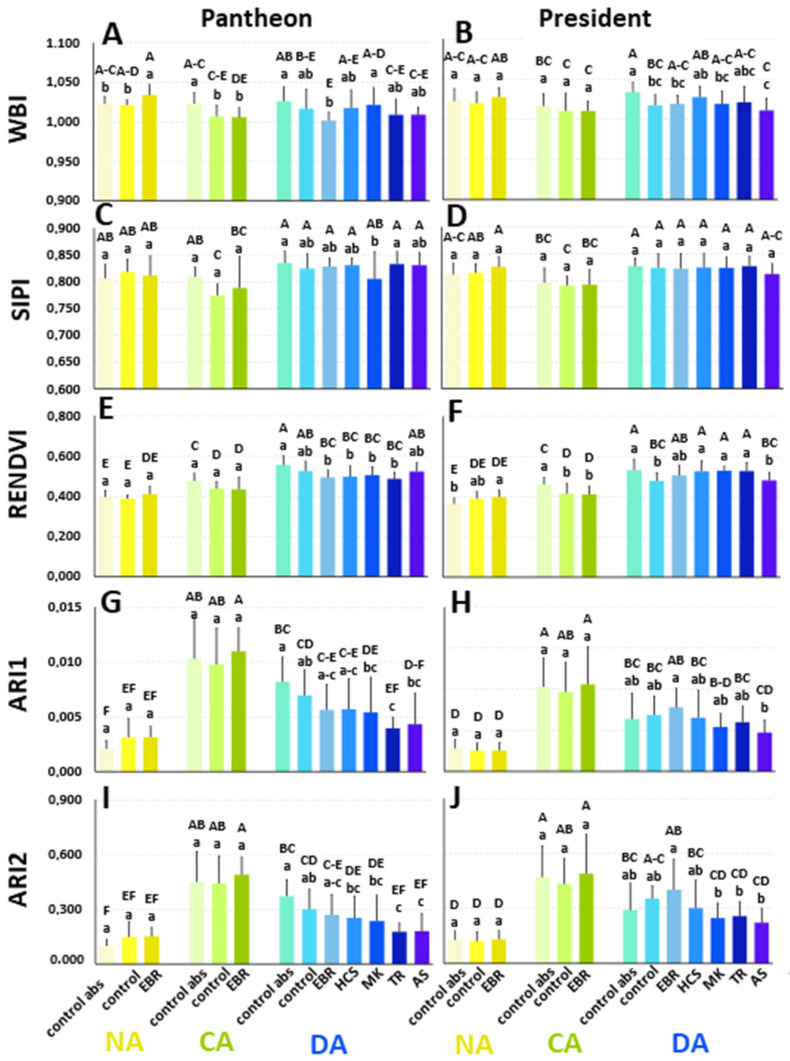
The leaf reflectance parameters of the leaves of the non-acclimated (NA), cold-acclimated (CA), and deacclimated (DA) oilseed rape cultivars Pantheon (**A**,**C**,**E**,**G**,**I**) and President (**B**,**D**,**F**,**H**,**J**). WBI—Water Band Index (**A**,**B**); SIPI—Structure Insensitive Pigment Index (**C**,**D**); RENDVI—Red-Edge Normalised Difference Vegetation Index (**E**,**F**); ARI1—Anthocyanin Reflectance Index 1 (**G**,**H**); ARI2—Anthocyanin Reflectance Index 2 (**I**,**J**). Control abs—untreated plants; control—plants treated with DMSO (a solvent of the tested steroids). The other objects represent plants that had been sprayed with brassinosteroids (EBR—24-epibrassinolide; HCS—28-homocastasterone), brassinosteroid analogues (MK—MK-266; TR—triolon), and the regulator Asahi SL (AS). Mean values indicated by the same letters did not differ significantly according to Duncan’s test (*p* < 0.05). Lowercase letters—comparisons between treatments within a specific group (NA, CA, and DA plants); capital letters—comparisons between the treatments of plants of all three groups (NA, CA, and DA plants).

Based on the reflectance curves, the following parameters of reflectance were calculated: the Water Band Index (WBI), Structure Insensitive Pigment Index (SIPI), Red-Edge Normalised Difference Vegetation Index (RENDVI), Anthocyanin Reflectance Index 1 (ARI1), and Anthocyanin Reflectance Index 2 (ARI2) (Figure 6). Additionally, the Triangular Vegetation Index (TVI), Simple Ratio Pigment Index (SRPI), Normalised Difference Vegetation Index (NDVI), Greenness Index (G), and Carotenoid Reflectance Index 1 (CRI1) were also calculated (Figure S5). As for the values of additionally calculated parameters (TVI, SRPI, NDVI, and CRI1), there were no significant changes between NA, CA, and DA plants. There was also no influence of the tested regulators (Figure S5A–F,I,J). An exception was the Greenness Index, which slightly decreased in CA and DA plants. A slight effect of the steroids was observed in the DA group. For example, TR increased the G value in Pantheon plants in comparison to both controls (Figure S5G,H).

The Water Band Index (WBI) provides information about the amount of water in the tested leaf tissue. For the control abs of NA Pantheon plants, the WBI reached a value of 1.023 (Figure 6A). It increased after cold acclimation and remained at the same level after deacclimation. The application of DMSO (control) decreased the WBI only after cold acclimation. EBR increased the values of the WBI only in the NA plants. In the CA and DA plants, EBR had no effect compared to the DMSO-treated control. The other regulators—HCS, MK, TR, and AS, which were applied only to the DA plants—did not affect the WBI.

In the cultivar President, the WBI that was characteristic of the NA (control abs) plants was 1.018, and it remained unchanged in the CA and DA plants (Figure 6B). The application of DMSO did not affect the WBI in the NA and CA plants; however, it significantly decreased the WBI in the DA plants. There was no effect of EBR in the NA and CA plants. In the DA plants, the application of BRs, BR analogues, and AS did not affect the WBI when compared to the control with DMSO.

The Structure Insensitive Pigment Index (SIPI) generally characterises the carotenoids/chlorophyll *a* ratio. In the cultivar Pantheon, there were no significant differences in the SIPI values between the NA, CA, and DA plants (control abs) (Figure 6C). The application of DMSO (control) did not affect the SIPI values in the specific groups of the NA, CA, and DA plants compared to the control abs plants. There were no significant effects of any of the tested regulators on the SIPI values.

In the NA President plants, the value of the SIPI was 0.81, and it did not significantly change after cold acclimation and deacclimation in the plants of the control abs (Figure 6D). The DMSO in the working solutions did not affect the SIPI values in the sprayed control plants. There was also no effect of the BRs and BR analogues on the SIPI values.

RENDVI is a parameter that is usually used to indirectly estimate the photosynthetic capacity and net primary productivity. In our study, the value measured for the NA control abs (Pantheon plants) reached 0.396 (Figure 6E). After cold acclimation, it increased significantly, by 20%. After deacclimation, there was a further increase of 17% in the value of the RENDVI. The application of the working solutions containing DMSO did not have any effects. EBR did not affect the RENDVI in the NA and CA plants; however, in the DA plants, the use of EBR caused a decrease in the RENDVI value when compared to the control abs. The application of the other regulators did not change the RENDVI values when compared to the control plants.

In the cultivar President, the RENDVI value that was measured for the NA control abs plants reached a value of 0.362. In the CA plants (control abs), the RENDVI value increased significantly, by 27% (Figure 6F). After deacclimation, there was another increase in the RENDVI (16%). The application of a working solution containing DMSO in the NA plants did not affect the RENDVI. In the CA and DA plants, DMSO decreased this index by about 10% compared to the control abs. The application of EBR and the other regulators, except for AS, did not affect the RENDVI value. AS caused a significant decrease in the RENDVI compared to the control abs.

ARI1 and ARI2 are parameters that reflect the presence of anthocyanins in plants. In the NA oilseed rape cv. Pantheon, the value of ARI1 for the control abs plants was 0.0021 (Figure 6G). After cold acclimation, ARI1 increased significantly, by about fourfold, while after deacclimation this value decreased, although it did not reach the value that was characteristic of the NA plants. The same trend was observed for ARI2 (Figure 6I). Spraying with DMSO (control) did not affect the ARI1 and ARI2 values in the NA, CA, and DA plants. EBR also did not affect ARI1 and ARI2 in the NA and CA plants. The application of all of the regulators to the DA plants resulted in a decrease in the ARI1 and ARI2 values, particularly in the cases of TR and AS.

In the NA control abs of President plants, the ARI1 value reached 0.0028 (Figure 6H). In the CA plants, the ARI1 value increased significantly, by almost threefold. After deacclimation, ARI1 decreased by about 40% compared to the CA plants; however, it did not reach the level that was characteristic of the NA plants. An identical trend was observed for ARI2 (Figure 6J). The application of DMSO did not affect ARI1 and ARI2 in the NA, CA, and DA plants. The application of EBR did not change the ARI1 value in the NA and CA plants. In the case of the DA plants, EBR had practically no effect. The use of the other regulators in the DA plants caused only a minor decrease in the values of the calculated parameters.

## 3. Discussion

### 3.1. The Frost Tolerance of Plants and the Modulation of the Properties of Their Membranes by BRs and BR Analogues

Basal frost tolerance characterises non-acclimated plants and enables them to survive slight frost, e.g., −1 to −3 °C, usually without injuries or with some small injuries [11]. On the other hand, it is commonly known that, after cold acclimation, winter cultivars become more frost-tolerant. This phenomenon was also observed in our experiment. The attempt to improve the frost tolerance of cold-acclimated plants by applying EBR was only successful for President compared to the absolute control and the DMSO-treated control plants. In Pantheon, the negative effect of DMSO was, in fact, reduced by the application of EBR, but in the end, the frost tolerance of the EBR-treated plants was similar to that of the absolute control. To conclude, in the case of the cold-acclimated oilseed rape plants, the effect of EBR seemed to be cultivar-dependent. This is in agreement with earlier studies on cold-acclimated winter wheat [28] and cold-acclimated perennial ryegrass (*Lolium perenne* L.) [32], where the application of EBR reduced frost injuries in a cultivar-dependent manner. In our experiment, EBR was also applied in order to improve the basal tolerance of oilseed rape (non-acclimated plants); however, it had no effect. We somehow expected a protective effect of EBR against frost in non-acclimated and cold-acclimated oilseed rape based, among other thing, on earlier studies of [30] devoted to *A. thaliana*—a species of the same family as oilseed rape. Studies on mutants have proposed that brassinosteroids participate in the control of the basal and acquired freezing tolerance of *A. thaliana*. The BR-deficient mutants of *A. thaliana* were hypersensitive to freezing stress, whereas the activation of BR signalling increased their freezing tolerance both before and after cold acclimation [30]. Our results only confirm that the effects that were observed after the exogenous application of the hormone are different from the effects that are observed in mutants with hormonal disturbances. A similar situation was observed in the case of deacclimated plants. The protective effects of selected growth regulators, BRs, or BR analogues against frost in deacclimated plants are reported here for the first time. However, the results are somewhat contradictory to earlier studies that were carried out on deacclimated barley mutants with disturbances of the BR biosynthesis or signalling [16]. Lower contents of endogenous BRs or defects in the BR receptors were accompanied by a better frost tolerance of deacclimated barley mutants. The exogenous application of BRs rather increases the natural concentrations of BRs [33]. The reasons for the contradictions/inconsistencies between the results obtained for mutants and for plants exogenously treated with steroids are complex. Among other factors, they are connected to the additional influence of various factors that will be discussed in a further part of the text.

In addition to the observed cultivar-dependent effects and some structure-dependent activity of the steroids that were used, the intensity of frost was also significant. A positive effect of EBR was observed in the DA plants at a temperature of −6 °C, although that effect disappeared at lower temperatures (−9 and −12 °C), where injuries to the plants were generally too strong to be alleviated by the application of the regulators.

Another factor that seriously disturbed the assessment of the physiological effects of the tested BRs and BR analogues was the presence of the solvent DMSO in the working solutions. It is known that hormone solvents can affect the metabolism of plant cells [34]. This was the situation that most clearly occurred in the DA President plants, where, after the frost test at −9 °C, it was observed that the plants that had been sprayed with DMSO had better frost tolerance than the control abs plants; therefore, the effect of the steroids was not proven. From the point of view of agricultural practice, the effects of the applied regulators should be proven relative to an absolute control with a simultaneous lack of positive effects (or only weak positive effects) being generated by working solutions containing solvents. When it would be recommended that brassinosteroids be used in fields to protect plants against frost, solvents that are less active in plants should probably be sought for commercial preparations.

Finally, the effects of the tested regulators should be considered relative to the extent to which these compounds penetrate the tissue—i.e., pass through the cell walls and membranes—after being sprayed on the leaf surface. The structure of the cell wall and cell membranes depends on the thermal conditions in which plants grow. There are significant differences in the cell wall structure between NA, CA, and DA plants of *A. thaliana* [35]. On the other hand, the fluidity of membranes is different in plants that are growing at higher temperatures [36,37] than in plants that are growing at lower temperatures [38,39,40,41]. Because the biological membranes of cells alters their lipid composition in order to acclimate the plant to lower/higher temperatures, this “rearrangement” of the composition results in a different membrane fluidity, which consequently causes a varied localisation and interaction of different compounds. According to [4], steroids that have different chemical structures interact differently with lipid monolayers with a different degree of fluidity and different lipid components, such as phospholipids and galactolipids.

Because EBR and HCS have already been proven to be modulators of the physicochemical properties of membranes [4,42], in the current work, we only conducted supplementary studies for two BR analogues. The analysed MK-266 and triolon have basic chemical structures that are typical for steroids. The strongly hydrophobic character of the molecular skeleton favours localisation within membranes [43]. Indeed, studies that have been conducted in model systems that differ in the degree of lipid saturation have revealed changes that occur at the physicochemical and structural levels of membranes due to treatment with MK-266 and triolon. Specifically, an increase in the A_lim_ parameter with a rising concentration of the hormone in the examined monolayers might indicate their interaction/localisation within the model membrane. An analogous trend in membrane modifications had previously been observed for other steroids [42,44]. An increase in the values of the A_lim_ parameter, which provides information about the fluidity of the monolayer and was induced by BR analogues in the current work and by EBR in earlier studies, could be one of the mechanisms responsible for improving the frost tolerance of the tested plants. Because of these mechanisms, the proteins that are present in the membranes can function properly despite any changes in temperature and alterations in the mechanical properties of the membranes. However, it should be remembered that, in our Langmuir studies, only defined (and somewhat higher) concentrations of steroids were active. In conclusion, the fluidity of the membranes, which was increased by the analogues, would be important for frost tolerance, but this is only one of the possible mechanisms. Generally, the weaker effects (on frost tolerance) that were obtained for the plants that had been sprayed with MK rather than with TR or EBR could be a result of differences in chemical structure between these steroids. In MK, an additional ring is attached to a carbon chain at position C17 of the sterane skeleton. This could be a reason for the weakened physiological activity of MK (see also: net photosynthesis in Figure 4A). However, this hypothesis should be confirmed in further research.

In the context of the importance of membrane fluidity for the effects that are caused by steroids, it is worth commenting on the results of experiment 2, which included a simplified laboratory test that was performed on cut leaves, and in which the effects of steroids were investigated by determining the electrolyte leakage after the leaf tissue was frozen. Measuring the electrolyte leakage is a method that enables the condition of cellular membranes—specifically, their permeability—to be assessed [45]. In cold-acclimated *A. thaliana*, when the freezing tolerance increased, the electrolyte leakage decreased compared to the non-acclimated plants [46]. In our studies, this simple phenomenon was confirmed in the control abs of the non-acclimated and cold-acclimated leaves. On the other hand, the decreased electrolyte leakage that was observed here in the deacclimated and frozen leaves (vs. the cold-acclimated leaves) was surely connected to the shorter duration of freezing that was used for the leaves of the DA plants. However, the most important observation in experiment 2 was that the application of steroids increased the membrane permeability of the detached leaves. This effect was particularly visible for all of the steroids that were applied to the cold-acclimated plants (Table 2). As for the leaves of the non-acclimated and deacclimated plants, when the steroids were applied to the plants that were growing at a higher temperatures, the effect was only statistically proven for MK and TR. In this context, earlier studies of the interactions of HCS and castasterone with model membranes, which also showed a dependency of the effects caused by these steroids on the temperature at which they were applied to the lipid monolayer, are also interesting [42]. Specifically, these natural brassinosteroids increased the fluidity of the model membranes when they were applied at 20 °C, but they decreased it when they were applied at cold temperatures (10 °C). Thus, theoretically, in experiment 2, the application of natural BRs (such as EBR or HCS) in the final week of cold acclimation could “stiffen” the membranes, thus making the leaves more susceptible to freezing temperatures. In contrast to the natural BRs, the BR analogues increased the membrane permeability on a more or less similar level in the leaves of the NA, CA, and DA plants (Table 2). Therefore, it is possible that this effect is also structure-dependent. The explanation of the observed phenomenon requires further studies, but the preliminary conclusions that can be drawn here could have a practical aspect. The moment that a steroid is applied, particularly natural BRs, and the specific composition/fluidity of the cell membranes at that time should not be neglected. It seems that in order to improve the frost tolerance of plants that have been subjected to cold acclimation, the steroid (EBR, HCS) should instead be applied before the cold acclimation, as was the case in experiment 1. In turn, in order to improve the frost tolerance of plants that are at risk of deacclimation, natural steroids could be administered during deacclimation, but detailed systematic applied studies are required.

### 3.2. Model of the Changes in the Accumulation of the Putative Brassinosteroid Receptor (BRI1) and the Accumulation of the Transcripts of the COR and SERK Genes

To the best of our knowledge, the presence of the putative BRI1 protein (brassinosteroid receptor) in oilseed rape is reported for the first time in this work. The accumulation of this protein decreased after cold acclimation, which is consistent with a decrease in the accumulation of the *BRI1* transcript [11]. It is worth emphasising that this phenomenon was characteristic of almost all of the studied cultivars ([11]; Figure 2 and Figure S4). The increased content of putative BRI1 that was observed in tested cultivars after deacclimation was accompanied by a higher abundance of the *BRI1* transcript in only two of them—President and Feliks [11]. An earlier analysis of endogenous BRs revealed significant differences in the contents of specific BRs between the four tested cultivars, and the pattern of changes was not very clear during cold acclimation or after deacclimation [11]. Generally, cold acclimation rather increased the concentrations of some of the BRs in oilseed rape. According to the literature, at higher concentrations, the BR content has stress-protective functions while at lower concentrations it has growth-promoting functions [47]. Thus, the increase in the concentrations of some of the BRs in cold could be expected and seemed to be justified. After deacclimation, a decrease can be observed in some of the BRs [11]. A lower content of BRs, which have growth-promoting activity [47], could then be somehow accompanied by a resumption of growth after deacclimation. The higher abundance of the receptor protein (despite a decrease in some of the BRs after deacclimation) could mean a strong signal transduction towards the resumption of growth that is induced by these growth-promoting steroids, which is unfavourable from the point of view of maintaining frost tolerance. Additionally, this would be consistent with the hypothesis of [47] that, at higher concentrations, BRs have stress-protective functions, and that the growth-promoting effects of BRs occur at lower concentrations because, as our results indicate, this might result from an increased level of the receptor protein.

In terms of stress-protective functions, it is known that BRs can affect, e.g., the COR protein expression [30]. In our study, we observed some agreement between the changes in the BR contents [11] and *COR* expression—an increase in those parameters during cold acclimation and a decrease after deacclimation.

The increased expression of the *SERK* genes, including *SERK1* and *SERK2,* was previously observed under abiotic stress conditions such as salinity stress in barley [48]. Moreover, an increased expression of *AcSERK2* was observed at a low temperature (4 °C) in *Ananas comosus* [49]. However, our results are more in line with the findings of [50], who observed a decreased expression of *DlSERK1* in *Diospyros lotus* under low-temperature treatment, as we also observed in CA oilseed rape. Generally, SERK1 is involved in BR signal transduction [51]; in our studies, the lower level of the *SERK1* transcript (but not *SERK2*) in the CA plants was accompanied by a decrease in the accumulation of the putative BR receptor (BRI1). The opposite effect was observed in the DA plants, which might confirm the role of BRI1 together with the SERK1 protein in the resumption of growth as an effect of a longer exposure of plants to higher temperatures (during deacclimation).

### 3.3. The Effects of BRs and BR Analogues on Photosynthesis

Regarding the light reactions of photosynthesis, PSII efficiency was assessed based on the chlorophyll *a* fluorescence measurements. Similar to our previous studies [11], there were some slight differences in the values of the parameters that describe PSII efficiency in both cultivars, and there were more significant changes between the groups of NA, CA, and DA plants. Generally, PSII efficiency, which is expressed by yield/flux ratios (φ_Po_, ψ_o_, φ_Eo_), was lower in the cold-acclimated plants. This effect was more visible in the cultivar President but was weaker in Pantheon, where only one of the three calculated parameters was lower in cold. The fact that cold acclimation decreases the efficiency of PSII has been well studied. The cold acclimation of barley at 5 °C (three weeks) resulted in a decrease in the maximum quantum efficiency of the PSII photochemistry (F_v_/F_m_ parameter), as well as a decrease in the general PSII efficiency, described as the P.I._ABS_ index [52]. In cold-acclimated *Avena sativa* L. (4/2 °C d/n, four weeks), the values of the maximum quantum efficiency of the PSII photochemistry (F_v_/F_m_) decreased [53]. The cold acclimation of oilseed rape also decreased the maximal fluorescence (F_m_) [11] and F_v_/F_m_ values [15]. After a period of cold, an increase in the temperature (during deacclimation) increases PSII efficiency. This effect was observed for both cultivars in our experiment, and it is in agreement with previous studies [11].

In our studies, calculation of yield/flux ratios showed that BRs had no effect on PSII efficiency in the NA, CA, or DA plants of oilseed rape. However, some effect of BRs was expected because, according to the literature, in cereals, BRs can limit the effects of low temperatures on the light reactions of photosynthesis, and especially on the energy flow in PSII [29]. In a cold-acclimated, EBR-treated, frost-tolerant cultivar of winter rye, the energy flow from the photosynthetic antennas to the electron transport chain was more effective than in the untreated control plants, and the energy that was lost as heat was lower. Even though a moderately frost-tolerant cultivar presented slightly different patterns of changes, EBR still decreased the energy lost as heat [29].

In contrast to the light reactions of photosynthesis, the measurements of gas exchange clearly indicated that, in both cultivars, BRs had an effect on some of the parameters associated with the dark reactions of photosynthesis. The values of the most important parameter (P_N_), which provides information about the CO_2_ assimilation, were on a similar level in the NA and CA plants of both controls, which is in agreement with the earlier findings of [15]. On the other hand, ref. [29] observed that six weeks of cold acclimation of winter rye resulted in a higher activity of Rubisco (a CO_2_-binding enzyme), which might be associated with the increased need for sugar accumulation. Typically, during cold acclimation, the accumulation of soluble sugars increases in order to protect the cell sap from freezing [5]. This process is favourable for increasing frost tolerance, and it was also observed earlier by [15] for two cultivars of oilseed rape. In our experiment 1, in both cultivars of oilseed rape, EBR strongly increased the CO_2_ assimilation in cold, which was clearly accompanied by a lower intracellular concentration of CO_2_. According to the literature, the exogenous application of EBR on cold-acclimated winter rye also increased the activity of Rubisco and increased the accumulation of soluble sugars [29]. The application of EBR on cold-stressed maize seedlings resulted in increased amounts of sugars: glucose, starch, and sucrose [54]. Taken together, higher CO_2_ assimilation in EBR-treated oilseed rape growing in cold conditions could be accompanied by higher sugar production and could be responsible, as one of several possible mechanisms, for the better frost tolerance of cold-acclimated plants, especially in the cultivar President. A barely provable effect of EBR on the CO_2_ assimilation (P_N_) was visible in the non-acclimated plants, and the frost tolerance was also not affected by this hormone in these plants. In the deacclimated plants that had been treated with EBR and HCS, P_N_ increased, and here the explanation could still be the same as for CA plants. Better assimilation of CO_2_ may favour sugar production and could be one of many mechanisms that are beneficial for a better frost tolerance of steroid-treated plants. Regarding the BR analogues and their influence on the assimilation of CO_2_ in connection with the effects of these steroids on frost tolerance, some cultivar dependency was observed. In the DA President plants that had been treated with TR, higher assimilation of CO_2_ was still accompanied by increased frost tolerance. In the case of the MK-treated plants, there was no effect on the assimilation of CO_2_, and there was also no effect on their frost tolerance. In Pantheon, this connection was not so clear. As mentioned earlier, weaker activity of MK could be a result of differences in its chemical structure (Figure S7) in comparison to TR or to natural BRs. At position C17 of the sterane skeleton, MK has an additional ring attached through a carbon chain.

Generally, in our experiments, higher values of P_N_ were observed in the deacclimated plants than in the NA and, particularly, the CA plants. In our opinion, this is an effect that could be expected for photosynthesis at higher air temperatures, and which could be accompanied by a later resumption of growth (due to the need for the higher synthesis of the assimilates). In the literature, however, the opposite effects have been observed. In the studies of [55] in deacclimated oilseed rape plants of the winter cultivar Górczanski, there was decreased activity of the CO_2_-binding enzyme (Rubisco) compared to the cold-acclimated plants. The authors of [15] also reported a decrease in the P_N_ values in the DA cultivars of winter oilseed rape compared to the CA plants. The matter of regulation of photosynthesis in deacclimated plants could thus be more complex and requires further studies.

The other parameters of gas exchange—transpiration [E] and WUE—also exhibited specific dynamics and were different in the NA, CA, and DA plants of both cultivars. Transpiration was predictably lower in cold and increased at higher temperatures in NA and DA plants. The photosynthetic ratio of WUE, here understood as the ratio P_N_/E, is a parameter that generally provides information about the amount of carbon that is assimilated as biomass [56]. Because the values of transpiration were low in cold, while P_N_ was similar in NA and CA plants, the WUE ratio was also increased in cold in both cultivars. WUE decreased again after deacclimation relative to the intensified transpiration processes. Due to the especially high values of P_N_ in the CA plants that had been treated with EBR, the WUE was additionally increased in these plants compared to the CA control abs and the control.

To conclude, from a practical point of view, monitoring the gaseous exchange—especially changes in E and WUE (also in P_N_)–can provide good indicators that enable the moment that plants become deacclimated be recognised.

### 3.4. Leaf Spectral Properties and Their Usefulness for Detecting Deacclimation

No significant effects of the tested regulators on leaf spectral properties were observed. Measurements of leaf reflectance, however, appear to be a good non-invasive method that could enable the early detection of deacclimation in plants. According to the literature, the spectral properties of leaves might be an indicator of the stresses that affect plants [57]. Of particular interest is the reflectance at visible wavelengths of 400–720 nm, and in our study most of the changes were detectable within this range. From the point of view of the early detection of deacclimation, attention was paid to the reflection at 550 nm, which gave the first pick on the reflectance curve (Figure 5). This phenomenon was present independent of the cultivar or treatment (hormone EBR, control with DMSO). The NA plants were characterised by the highest values of reflectance at 550 nm, while lower values were observed for CA and the lowest for DA. Currently, in agriculture satellite imagery, the so-called “red-edge” region of reflectance (670–760 nm) is promoted for detecting plants’ stress [58]. The shift in the reflectance curve (red-edge spectral region) on the left side is interpreted as characterising stressed plants [58]. In our experiment, the curves that characterised the NA, CA, and DA plants were in the same place within the red-edge spectral region (Figure 5A–F). For this reason, we suggest that attention should be paid to the range of 520–650 nm, with particular emphasis on the peak at 550 nm, to assess the occurrence of deacclimation.

Based on the reflectance curve, various parameters and ratios can be calculated, e.g., WBI, SIPI, RENDVI, ARI1, and ARI2.

The common range of WBI for green vegetation is from 0.80 to 1.20 [59]. The results that were obtained for the Pantheon and President plants from all of the treatments had a WBI that ranged between 1.00 and 1.03. Those values are in agreement with [59] and did not mean a water deficit in the CA and DA plants, even though there were some significant changes between the BR-treated and control plants. The patterns of the changes in the values of WBI for the NA, CA, and DA plants of Pantheon and President were slightly different than the results that were obtained by [15] for different cultivars of oilseed rape. However, despite this, those values did not indicate a water deficit in the plant tissues. From a practical point of view of the non-invasive detection of deacclimation in plants, WBI does not seem to be useful.

The Structure Insensitive Pigment Index (SIPI) is a function of chlorophyll *a* and carotenoids [60]. In addition, the SIPI is an approach that minimises the confounding effects of the leaf surface and mesophyll structure in estimating the carotenoid/chlorophyll *a* ratio [60]. However, the SIPI is sensitive to the leaf water status, among a few other factors [61]. The values for the SIPI range from 0 to 2. The typical range for green vegetation is between 0.8 and 1.8 [60,61]. In our studies, the values ranged from 0.774 to 0.835. Only minor changes, which were mostly insignificant, were observed between the NA, CA, and DA plants, as well as after the steroid treatments. Similar to the WBI, from the practical point of view, the SIPI does not seem to be useful for the non-invasive detection of deacclimation in plants.

The Red-Edge Normalised Difference Vegetation Index (RENDVI) is a modification of the Normalised Difference Vegetation Index (NDVI), which is a broadband index that is associated with green biomass and has been used to indirectly estimate the photosynthetic capacity and net primary productivity using the reflectance measurements along the red-edge [62]. In our experiment, this index increased with the duration of vegetation, independent of the cultivar.

In contrast to the RENDVI, the values of the Greenness Index (G) decreased during plant growth, and they were highest in the NA plants and lowest after deacclimation. G is slightly correlated with the contents of chlorophyll and other photosynthetic pigments [63]. From a practical point of view, for the non-invasive detection of deacclimation in plants, both the RENDVI and G could be useful. However, due to the changes in the values of the RENDVI and G over time, we cannot exclude the possibility that these values change as a result of the progress of plant growth/development. Hence, this issue requires further studies, where plants grown in parallel are not acclimated throughout the entire experiment or are cold-acclimated for a longer period than in the current study (i.e., six weeks).

The Anthocyanin Reflectance Index 1 (ARI1) enables the accumulation of anthocyanins to be estimated even in small amounts in intact senescing and stressed leaves [64]. The common range of this index is from 0.001 to 0.1. Another index that reflects the anthocyanin content is the Anthocyanin Reflectance Index 2 (ARI2), which is a modified anthocyanin reflectance index that is less dependent on leaf thickness and density, and is able to detect higher concentrations of anthocyanins in vegetation [64]. In our study, both of those indices exhibited a tendency to increase in the CA plants and decrease in the DA plants. This is a similar tendency to the one that was observed for ARI, calculated according to the equation for ARI2, in the earlier studies of [15] on two other cultivars. Changes in the values of the parameters from the ARI group are also supported by fact that, under abiotic stresses such as a low temperatures, plants accumulate an increasing number of anthocyanins, and this phenomenon has been observed in many plant species—for example, *A. thaliana* and apple (*Malus domestica*) [65,66]. Thus, an increase in the ARI values in cold, along with their later decrease at higher temperatures (deacclimation), is in agreement with these data. Therefore, it can be concluded that the ARI parameters (1 and 2) could be a useful indicator for assessing a plant’s deacclimation using non-invasive measurements.

To summarise, attempting to find methods that could enable us to detect deacclimation seems to be increasingly important today. Due to climate change and the more frequent changes in weather patterns, the phenomenon of deacclimation threatens many winter crop plant species, especially when sudden frost occurs after a warm period of deacclimation. Earlier detection of deacclimation using non-invasive techniques, such as measuring photosynthesis or leaf reflectance properties, could enable some of the regulators that at least limit the possibility of frost injury after deacclimation to be used. In our research, the results were obtained in control conditions; thus, it is advisable that the pattern of the changes of specific parameters be confirmed in open-field conditions. However, the positive information is that non-invasive methods for the early detection of deacclimation could also be useful on a large scale by using drones or satellites to detect any changes in reflectance [58,67] or changes in chlorophyll *a* fluorescence [68].

Concluding remarks are given in Section 5.

## 4. Materials and Methods

### 4.1. Plant Material

Experiment 1 was conducted on two winter cultivars of oilseed rape (*Brassica napus* L. var. *napus* L.): President and Pantheon. Both cultivars are hybrid cultivars (F1), and the seeds were obtained from Saatbau, Środa Śląska, Poland. The plant material in the experiments were analysed on these cultivars, while exceptionally, for the analysis of the accumulation of the protein BRI1, three additional cultivars of oilseed rape were included in the experiment—the winter cultivars Rokas and Bojan, and the spring cultivar Feliks. In experiment 2, only the President cultivar was chosen.

### 4.2. Experimental Design

The experimental design consisted of a few separate experiments (1, 2, and 3), which are a continuation of previous research devoted to the deacclimation-induced biochemical/physiological changes in oilseed rape [11,15,18].

#### 4.2.1. Experiment 1

In experiment 1, the main aim was to verify the hypothesis that the selected BRs and BR analogues might improve the frost tolerance of oilseed rape, especially the frost tolerance after deacclimation.

The experimental design was similar to the model that was described in detail in our earlier article [11]. The detailed number of plants used in the experiment, i.e., the number of plants in the pods/treatments, is given there [11]. Growth conditions such as light source, light spectrum, and intensity are also given in [11]. The experimental design is presented in Figure S6. Briefly, the seeds of oilseed rape were germinated on Petri dishes at 24 °C in darkness for three days. The seedlings were transferred into pots with soil and cultured at 20 °C for four days, and then at 17 °C for 17 days. Earlier, at day 21 of vegetation, the pots with plants were divided into three groups.

In the first group, referred to as non-acclimated plants (NA), the 21-day-old plants were divided into the next three groups: NA plants that had been sprayed with 24-epibrassinolide (EBR), NA plants that had been sprayed with DMSO—a solvent of EBR (control)—and NA plants that had not been sprayed and served as an absolute control (control abs). Three days after spraying, the plants were exposed to frost (−3 °C), and the frost tolerance of the NA plants—so-called basal frost tolerance—was estimated based on the regrowth of the plants (for details, see Section 4.3.1).

The second group of 21-day-old plants continued growing at 17 °C for three days. Then plants were pre-hardened for six days and then cold-acclimated at 4 °C for three weeks (days 31–51 of vegetation). This group was referred to as cold-acclimated plants (CA). One day before beginning the process of cold acclimation, the plants were sprayed with EBR or DMSO, and a group of unsprayed plants was left as an absolute control. All of the 51-day-old CA plants, after pre-hardening and cold acclimation, were exposed to frost (−13 °C), and then their frost tolerance was estimated based on the regrowth of the plants (for details, see Section 4.3.1).

The third group of 21-day-old plants continued growing at 17 °C for three days. Next, the plants were pre-hardened, cold-acclimated as described above, and then were also deacclimated at 16/9 °C between days 52 and 58 of vegetation. This group was referred to as deacclimated plants (DA). On day 55 of vegetation, the plants were divided and then sprayed with the following compounds: brassinosteroids—EBR and 28-homocastasterone (HCS); and brassinosteroid analogues—triolon (TR) and MK-266 (MK). The plants that had been sprayed with DMSO served as controls. Moreover, the group of plants in this group was also sprayed with a commercial preparation, Asahi SL, (Agrecol, Wieruszów, Poland) (AS). The plants that had not been sprayed served as absolute controls. At day 58 of vegetation (the seventh day of deacclimation), the DA plants had been exposed to frost at −6, −9, and −12 °C, and then their frost tolerance was estimated based on the regrowth of the plants (for details, see Section 4.3.1).

In all of the cases, the concentration of the tested steroids, BRs, and their analogues was 0.5 mg/L. The working solutions were prepared based on stock solutions of these steroids that had been dissolved in DMSO as a solvent (2 mg/0.5 mL of DMSO). This is why the control plants were sprayed with solutions that contained an adequate concentration of DMSO in water. In the case of Asahi SL, its concentration was adjusted based on the manufacturer’s protocol. In all of the cases, about 10 mL of the working solution was spread out over all of the plants growing in one pot. The BR analogues were generously provided by the Laboratory of Growth Regulators, Faculty of Science, and Institute of Experimental Botany of the Czech Academy of Sciences (Olomouc, Czech Republic). The EBR and HCS were purchased from Sigma Aldrich, Poznań, Poland.

The effects of BRs and BR analogues on photosynthesis were studied in this experiment. Non-invasive measurements of photosynthesis (PSII efficiency, gas exchange) and the leaf spectral properties (leaf reflectance) were always taken for the leaves of the plants from all of the groups (NA, CA, and DA). In NA and DA plants, measurements were taken one day before the frost test. In CA plants, measurements were taken two days before the frost test. The non-invasive measurements were always taken from the best-developed leaf in the leaf rosette of the plants. Moreover, before the frost tests, samples were taken for analyses of the accumulation of the protein BRI1 (brassinosteroid membrane receptor), the accumulation of transcripts of genes encoding the proteins that participate in BR signalling (*SERK1* and *SERK2*), and the accumulation of the transcript of *COR*. *COR* is a BR-regulated gene that is connected to the acclimation of plants to low temperatures. Samples were also taken from the best-developed leaves and immediately frozen in liquid nitrogen. The exact times of the measurements and sampling are given in Figure S6.

#### 4.2.2. Experiment 2

In experiment 2, the aim was to answer the question of whether/how BRs and their analogues modify the membrane permeability that was measured for the leaves of NA, CA, and DA plants that had been exposed to frost.

Briefly, this experimental design was similar to the one described in [11] and Section 4.2.1, with modifications (see also: Figure S6). In comparison with experiment 1, the spraying of plants with steroids (EBR, HCS, MK, TR) was carried out in each group of plants (NA, CA, and DA). Plants that had been sprayed with a solution containing DMSO (a solvent for steroids) served as controls, while plants that had not been sprayed served as absolute controls. Further, in comparison with experiment 1, the moment of spraying of a group of CA plants was changed, and spraying took place seven days before the end of a period of cold acclimation. The regulator Asahi was not tested in this experiment.

Two days after being sprayed, the best-developed leaves were cut off from the NA and DA plants. Next, the leaves were placed in Petri dishes (one leaf/one dish) and exposed to frost. In the case of CA plants, the leaves were cut off three days after spraying. In all three groups (NA, CA, and DA), after frost exposure, the electrolyte leakage was measured. Electrolyte leakage provides information about any changes in membrane permeability. A detailed description of the frost test and the measurements of electrolyte leakage is presented in Section 4.3.2.

The preparation of the solutions for spraying was the same as those described in experiment 1, and the concentrations that were used were also the same. The temperature of the frost test and the duration of keeping the leaves in the frost chamber were established based on preliminary trials.

#### 4.2.3. Experiment 3

The main aim of experiment 3 was to describe the interaction of two BR analogues, triolon and MK-266, with defined model lipid systems that have been described previously. The chemical structure of those analogues is given in Figure S7. Similar to the study of [42], in order to assess the impact of the tested MK-266 and triolon, lipids with varying saturation levels but with unchanged polar components were selected for the Langmuir monolayer model studies. 1,2-Dilinolenoyl-sn-glycero-3-phosphocholine (PC 18:3) was selected to represent unsaturated lipids, while 1,2-dipalmitoyl-sn-glycero-3-phosphocholine (PC 16:0) was selected as a lipid that only contains saturated fatty acids. The mixed system of PC (18:3) + PC (16:0) at a 1:1 molar ratio was selected as being equivalent to natural membrane systems, in which saturation is regulated by the proportions of the saturated and unsaturated acids. The research system that was used, in which the polar parts remained constant, permitted a clear analysis of any modifications in the impact of the tested compounds on the membranes, which resulted solely from changes in the fatty acid saturation.

The lipid solutions were prepared by dissolving synthetic PC 16:0 and PC 18:3 (Avanti Polar Lipids, Alabaster, Alabama, USA) in chloroform (Avantor, Gliwice, Poland) to achieve a final concentration of 1 mg/mL. Then, the two-compound lipid solution, which was represented by the saturated (PC 16:0) and unsaturated (PC 18:3) hydrophobic parts, was mixed at a molar ratio of 1:1 (PC 16:0 to PC 18:3). The triolon and MK-266 hormones were dissolved in chloroform. The final concentration was 1 mg/mL, with a small volume of methanol added to preserve solubility. Next, the solutions were mixed with the lipids at the following molar ratios (lipid-to-hormone)—4:1, 8:1, and 16:1.

### 4.3. Measurements

#### 4.3.1. Estimation of the Freezing Tolerance of Whole Plants in Pods

The temperatures for the freezing tolerance testing in experiment 1 were selected based on the authors’ previous experience [11] and were matched to the predicted frost tolerance of particular plant groups (NA, CA, and DA). The temperatures were as follows: the non-acclimated plants were tested at −3 °C, the cold-acclimated plants were tested at −13 °C, and the deacclimated plants were tested at −6 °C, −9 °C, and −12 °C. Technical details of reaching given temperatures below 0 °C are given in [11]. After the freezing test, the plants were transferred to a greenhouse with the temperature set at about 12 °C (natural light, November/December, Poland—eastern EU region). Two weeks later, the plant survival rate was estimated based on the visual score. The detailed description of the visual score is provided in our earlier article [11]. Briefly, the notes ranged between 0 and 7 points, where a 0–1 point score indicates a dead plant with no signs of leaf regrowth, and a 6–7 point score indicates a plant with few or no symptoms of injury visible on leaves. The frost test was carried out on 15 plants from each group: NA, CA, and DA plants.

#### 4.3.2. Measurements of Conductivity (Electrolyte Leakage) of the Detached Leaves

The leaves were cut, placed in sterile Petri dishes (⌀ 85 mm; one leaf/dish), and frozen at −18 °C in a freezing chamber for a duration that was selected based on previous testing and optimisation: 1 min 50 s, 4 min 30 s, and 2 min 30 s for NA, CA, and DA plants, respectively. Attempts were made to select the freezing time so that the leaves were only slightly injured. The analysis of electrolyte leakage, providing information about changes in the cell membrane permeability, was performed as described in [27,69], with necessary modifications. After the leaves were frozen in open Petri dishes, they were covered with redistilled and deionised water (20 mL per dish). The water that was used was produced using an Elgastat Maxima purification system (Elga, High Wycombe, UK). The Petri dishes with leaves in the water were covered with lids and then left at a temperature of 20 °C. Conductivity was measured 3 h and 24 h after the moment of freezing. Electrolyte leakage measurements were taken using a pH/conductivity meter (CPC-502, Elmetron, Zabrze, Poland). For each group of plants (NA, CA, and DA), and for each treatment (with BRs and BR analogues), ten replicates were made (10 different leaves).

#### 4.3.3. Langmuir trough Studies

The systems were prepared based on the method described in [42]. Single-compound solutions (PC 18:3 and PC 16:0) as well as mixed systems (PC 18:3 + PC 16:0 M:M molar ratio) were spread on an ultrapure deionised water subphase (HLP 5 apparatus “Hydrolab” (Poland)) to obtain the surface pressure isotherms. All of the systems were tested using a Langmuir trough (Minitrough, KSV, Finland) with a Pt-Wilhelmy plate. Each experiment was repeated three to five times to maintain a high level of repeatability and accuracy in the surface tension measurements (+/−0.1 mN/m). During the experiments, a constant temperature (20 °C) was thermostatically maintained.

Based on the Langmuir isotherms, three physicochemical parameters were calculated and determined (1) A_lim_—the area occupied by a single molecule in a maximum-packed layer, (2) π_coll_—the value of surface pressure at which the layer collapsed, and (3) the static compression modulus, which is defined as C_s_^−1^ = −(dπ/dlnA) and represents the mechanical resistance during mechanical compression and provides information about the layer stiffness.

#### 4.3.4. Analysis of the BRI1 Protein Accumulation

##### Measurements of the Protein Concentration in the Leaf Extracts

The samples that had been obtained from the leaves (1 g) were homogenised in liquid nitrogen and immediately extracted using 2.5 mL of extraction buffer containing 250 mM sucrose, 50 mM HEPES-KOH pH 7.5, 5% glycerol, 0.5% Triton X-100, 50 mM Na_4_P_2_O_7_, 1 mM Na_2_MoO_4_, 25 mM NaF, 2 mM DTT, and protease inhibitor cocktail tablets (Roche, Mannheim, Germany). The samples were centrifuged for five minutes at 38,030× *g* (MIKRO R, Hettich Centrifugen, Tuttingen, Germany). After centrifugation, the supernatant was collected, and the protein concentration was measured using 2-D Quant Kit (Cytiva, Marlborough, MA, USA) according to the manufacturer’s protocol, using a SynergyTM2 Multi-Detection Microplate Reader (BioTek, Winooski, VT, USA). Bovine serum albumin (BSA) (2-D Quant Kit, Cytiva, Marlborough, MA, USA) was used as the calibration standard. The analysis within experiment 1 was performed in three replications.

##### Analysis of the Accumulation of the BRI1 Protein in the Leaf Samples Using Immunoblotting

The samples were diluted in an SDS loading buffer at a ratio of 3:1. The SDS loading buffer contained 200 mM Tris pH 6.8, 400 mM DTT, 8% SDS, 40% glycerol, and 0.1% bromophenol blue. Protein denaturation was performed at 90 °C for 5 min. The same amounts (15 µg) of protein extracts were loaded and separated on 10% SDS-PAGE (1 mm polyacrylamide gel) according to the procedure described in [70]. After the proteins were separated, they were blotted onto low-fluorescence PVDF membranes (0.45 µm, Bio-Rad Laboratories, Inc., Hercules, CA, USA) (30 min, 25V/1A) using a semi-dry transfer (Bio-Rad Trans-Blot Turbo Transfer System, Bio-Rad Laboratories, Inc., Hercules, CA, USA). Then, the membranes were blocked with 5% low-fat milk diluted in a Tris-buffered saline/Tween (TSB-T) buffer (containing 0.9% NaCl, 10 mM Tris, and 0.5% Tween 20) for 1 h at room temperature (RT) with agitation. Next, the membranes were washed four times for five minutes with a TBS-T buffer at RT with agitation, and then they were incubated in the primary antibody (dilution: 1:2000; Anti-BRI1 (AS12 1859), Agrisera, Sweden) at 4 °C overnight. Next, the membranes were washed four times for five minutes with a TBS-T buffer, and then they were incubated with the secondary antibody (HRP-conjugated Goat anti-Rabbit IgG (H&L (AS09 602), Agrisera, Sweden) diluted with TBS-T buffer (1:5000) for 2 h at RT with agitation. The membranes were then washed three times for five minutes with a TBS-T buffer at RT with agitation, and the protein was visualised using the chemiluminescence method (AgriseraECL SuperBright (AS16 ECL-S), Agrisera, Sweden), with three-minute exposure duration of the membrane.

Dilutions of the antibodies were selected based on the previous optimisation process and the protocol of the manufacturer.

The optimisation of the analysis method included a trial on *A. thaliana* as a positive control in which the BRI1 accumulation was confirmed (AS12 1859 antibody manufacturer’s product information, Agrisera, Sweden). Simultaneously, on the same gel, samples of oilseed rape leaves were loaded. The obtained bands from samples of *A. thaliana* and oilseed rape were observed at the same level and were located above 130 kDa, as expected by the antibody producer (Agrisera). Thus, we claim that putative BRI1 protein was found in samples of oilseed rape. The results presented in this article were obtained using the same method.

Three independent replicates were performed. The densitometric analyses of the staining intensity of the visualised bands were performed to quantify the BRI1 protein content using ImageJ software version 1.53k (NIH, Bethesda, MD, USA). The averages are expressed as arbitrary units (A.U.) correlated with the area under the densitometric curves.

#### 4.3.5. Analysis of SERK1, SERK2, and COR14 Gene Expression

The quantitative real-time PCR analysis for the *COR14*, *SERK1*, and *SERK2* expression was performed using QuantStudio 3 (Thermo Fisher Scientific, Waltham, MA, USA). RNA extraction was carried out according to the manufacturer’s protocol using the RNeasy Plant Mini Kit (Qiagen, Hilden, Germany) from 50 mg of leaf tissue. The concentration and quality of each RNA sample were determined spectrophotometrically (Quawell, San Jose, CA, USA). Approximately 700 ng of RNA was subjected to a genomic DNA elimination, and a reverse-transcription reaction was performed immediately (QuantiTect Reverse Transcription Kit, Qiagen, Hilden, Germany) according to the manufacturer’s protocol.

The PCR primers for the *COR14*, *SERK1*, *SERK2*, and actin *Brassica napus* genes (Table 3) were designed using Primer Express Software v.3.0.1 (Applied Biosystems by Life Technologies, Foster City, CA, USA). The PCR amplifications were conducted in triplicate as described in [71], with a dissociation step to confirm the specificity of the reactions. The PCR data analysis was performed using QuantStudio Design and Analysis Software v.1.5.0. The relative standard curve method (Applied Biosystems) was used to calculate the relative gene expression. The *COR14*, *SERK1*, and *SERK2* expression levels were calculated relative to that of actin. The analyses were performed within experiment 1 in five biological repetitions.

#### 4.3.6. Chlorophyll A Fluorescence Measurements

To describe the efficiency of PSII, chlorophyll *a* fluorescence measurements were taken using a Plant Efficiency Analyser (PEA, Hansatech, King’s Lynn, UK). The leaves were covered with special clips for 30 min in order to adapt them to darkness. The following parameters were calculated based on the fluorescence (OJIP) curve as described in [31]: (1) φ_Po_—maximum quantum yield of the primary photochemistry (at t = 0); φ_Po_ = TR_0_/ABS = [1 − (F_o_/F_m_); (2) ψ_o_—probability (at t = 0) that a trapped exciton moves an electron into the electron transport chain beyond Q_A_^−^; ψ_o_ = ET_0_/TR_0_ = (1 − V_J_); and (3) φ_Eo_—the quantum yield of electron transport (at t = 0); φ_Eo_ = ET_0_/ABS = [1 − (F_o_/F_m_)]ψ). The measurements were taken within experiment 1. The measurements were always taken on the middle part of the best-developed leaf that was selected from the leaf rosette, and they were taken in ten replicates (each replicate was one leaf from different plants).

#### 4.3.7. Leaf Gas Exchange Measurements

Gas exchange was measured using an LCpro-SD infrared gas analyser (ADC BioScientific Ltd., Hoddesdon, UK), which automatically controlled the measurement conditions. The following parameters were measured: photosynthetic rate (P_N_), transpiration (E), stomatal conductance (g_s_), and intercellular concentration of CO_2_ (C_i_). The instantaneous water-use efficiency (WUE) was determined based on the quotient of the photosynthetic rate and transpiration (P_N_/E). The conditions in the measurement chamber were as follows: carbon dioxide concentration 470 μmol mol^−1^ air; temperature, air humidity, and PAR intensity equal to ambient. The measurements were taken in experiment 1 on the middle part of the first leaf from the top, in seven replicates.

#### 4.3.8. Leaf Spectral Properties (Leaf Reflectance Measurements)

To analyse the leaf reflectance in experiment 1, a CI−710S SpectraVue Leaf Spectrometer (CID–BioScience, Camas, WA, USA) was used. The following parameters of leaf reflectance were measured/calculated: (1) Water Band Index; WBI = (R900/R970) [59]. (2) Structure Insensitive Pigment Index; SIPI = (R800 − R445)/(R800 + R680) [60]. (3) Red-Edge Normalised Difference Vegetation Index; RENDVI = (R750 − R705)/(R750 + R705) [62]. (4) Anthocyanin Reflectance Index 1; ARI1 = (1/R550.8839) − (1/R700.9216). (5) Anthocyanin Reflectance Index 2; ARI2 = R801.1251∙((1/R550.8839) − (1/R700.9216)) [64] modified. Additionally, the following parameters were measured/calculated: (1) Triangular Vegetation Index; TVI = 0.5∙(120∙(R750 − R550)–200∙(R670 − R550) [72]. (2) Simple Ratio Pigment Index; SRPI = R430/R680 [60]. (3) Normalised Difference Vegetation Index; NDVI = (R800 − R680)/(R800 + R680) [73]. (4) Greenness Index; G = R554/R677 [63]. (5) Carotenoid Reflectance Index 1; CRI1 = (1/R510)–(1/R550) [74]. The measurements were always taken on the middle part of the best-developed leaf that was selected from the leaf rosette. Measurements were taken in experiment 1 in ten replicates, and each replicate was one leaf from the different plants.

### 4.4. Statistical Analyses

All of the statistical analyses were performed using Statistica 13.1. software (StatSoft, Tulsa, OK, USA). The results were analysed with ANOVA and Duncan’s post hoc test. Values that are marked with the same letters do not differ significantly (*p* < 0.05).

## 5. Conclusions

From the point of view of climate change, studies that are devoted to mechanisms of deacclimation are important. The knowledge that is acquired could help to counteract the adverse effects of warm breaks, e.g., in autumn/early winter, on winter crops that require the cold acclimation (hardening) process to survive temperatures below 0 °C in winter. Deacclimation generally induces a reversal of the cold-induced changes in the level of the putative brassinosteroid receptor protein (BRI1), the expression of BR-induced *COR*, and the expression of *SERK1*, which is involved in BR signal transduction. The dynamics of the changes in the accumulation of putative BRI1 and *SERK1* indicate that BRs might participate in the regulatory mechanisms that adapt plants to growth in different temperature conditions. The deacclimation-induced decrease in frost tolerance in oilseed rape could to some extent be limited by applying steroid regulators. The deacclimation in plants could be detected using non-invasive measurements such as leaf reflectance measurements (recommended parameters: ARI1 and ARI2). Characteristic changes in the leaf reflectance spectrum in the range 500–650 nm, could also be useful for the satellite monitoring of deacclimation.

## Figures and Tables

**Table 1:** The frost tolerance of the winter oilseed rape cultivars Pantheon and President, which were characteristic for non-acclimated (NA), cold-acclimated (CA), and deacclimated (DA) plants based on the regrowth scale. Control abs—untreated plants; control—plants treated with a water solution of DMSO (a solvent of the tested steroids). The other objects represent plants that had been sprayed with brassinosteroids (EBR—24-epibrassinolide; HCS—28-homocastasterone), brassinosteroid analogues (MK—MK-266; TR—triolon), and the regulator Asahi SL (AS). NT—not tested. Frost tests at −3 °C and −13 °C (NA and CA plants, respectively); DA plants were exposed to −6 °C, −9 °C, and −12 °C. Mean values ± SD that are indicated by the same letters did not differ significantly according to Duncan’s test (*p* < 0.05)—a comparison between plants treated with a different substance that had been grown in the same conditions; statistical analysis was conducted separately for the cultivars Pantheon and President.

Cultivar	Treatment	NA Plants; Frost: −3 °C	CA Plants; Frost: −13 °C	DA Plants; Frost: −6 °C	DA Plants; Frost: −9 °C	DA Plants; Frost: −12 °C
Pantheon	Control abs	6.86 ± 0.53 ^a^	3.75 ± 0.62 ^a^	3.14 ± 0.66 ^d^	2.20 ± 0.42 ^c^	2.00 ± 0.00 ^ab^
	Control	5.93 ± 1.10 ^b^	3.00 ± 0.00 ^b^	2.20 ± 0.42 ^e^	2.40 ± 0.55 ^c^	2.20 ± 0.42 ^ab^
	EBR	5.80 ± 1.08 ^b^	3.91 ± 0.94 ^a^	5.13 ± 0.83 ^a^	2.29 ± 0.73 ^c^	2.22 ± 0.44 ^a^
	HCS	NT	NT	3.53 ± 0.83 ^cd^	3.17 ± 0.58 ^b^	1.33 ± 0.78 ^cd^
	MK	NT	NT	4.21 ± 1.37 ^bc^	3.92 ± 0.67 ^a^	0.90 ± 0.57 ^d^
	TR	NT	NT	5.07 ± 1.27 ^a^	2.29 ± 0.91 ^c^	1.90 ± 0.32 ^ab^
	AS	NT	NT	4.54 ± 1.05 ^ab^	3.14 ± 0.53 ^b^	1.75 ± 0.62 ^bc^
President	Control abs	6.53 ± 0.64 ^a^	3.92 ± 0.90 ^b^	3.81 ± 0.98 ^c^	2.82 ± 0.40 ^b^	2.20 ± 0.42 ^a^
	Control	4.87 ± 1.96 ^b^	3.21 ± 0.43 ^c^	4.53 ± 1.06 ^bc^	3.73 ± 0.80 ^a^	2.00 ± 0.71 ^ab^
	EBR	7.00 ± 0.00 ^a^	4.69 ± 0.63 ^a^	5.60 ± 0.99 ^a^	3.91 ± 0.83 ^a^	2.00 ± 0.77 ^ab^
	HCS	NT	NT	5.47 ± 1.36 ^a^	3.50 ± 1.02 ^a^	1.92 ± 0.28 ^ab^
	MK	NT	NT	3.86 ± 0.95 ^c^	3.50 ± 0.52 ^a^	1.44 ± 0.73 ^b^
	TR	NT	NT	5.33 ± 1.18 ^ab^	3.83 ± 0.72 ^a^	1.92 ± 0.29 ^ab^
	AS	NT	NT	4.87 ± 1.06 ^ab^	3.33 ± 0.78 ^ab^	1.64 ± 0.67 ^b^

**Table 2:** Membrane permeability based on electrolyte leakage after 3 h and after 24 h from the moment of freezing in the leaves of non-acclimated (NA), cold-acclimated (CA), and deacclimated (DA) oilseed rape (President cultivar). H_2_O—redistilled/deionised water (without leaves); NFL—non-frozen leaves; Control abs—frozen leaves of untreated plants; control—frozen leaves of plants treated with a water solution of DMSO (a solvent of the tested steroids). The other objects represent frozen leaves of plants that had been sprayed with brassinosteroids (EBR—24-epibrassinolide; HCS—28-homocastasterone) and brassinosteroid analogues (MK—MK-266; TR—triolon). Mean values indicated by the same letters did not differ significantly according to Duncan’s test (*p* < 0.05). Comparisons within specific groups of plants (NA, CA, DA).

Growth Conditions and Freezing Time		Electrolyte Leakage after 3 h (µS)	Electrolyte Leakage after 24 h (µS)
NA 1 min 50 s	H_2_O NFL Control abs Control EBR HCS MK TR	1.4 ± 0.2 ^c^ 1.7 ± 0.3 ^c^ 53.8 ± 27.5 ^b^ 57.8 ± 35.7 ^b^ 49.9 ± 17.3 ^b^ 58.9 ± 24.9 ^b^ 108.4 ± 70.1 ^a^ 86.4 ± 28.8 ^ab^	1.5 ± 0.2 ^d^ 2.5 ± 0.7 ^d^ 128.2 ± 51.7 ^c^ 149.8 ± 77.8 ^bc^ 152.3 ± 33.8 ^bc^ 174.1 ± 61.1 ^abc^ 223.7 ± 96.3 ^a^ 210.7 ± 36.5 ^ab^
CA 4 min 30 s	H_2_O NFL Control abs Control EBR HCS MK TR	1.3 ± 0.1 ^d^ 2.5 ± 1.2 ^d^ 2.8 ± 1.2 ^d^ 17.1 ± 15.8 ^d^ 230.6 ± 44.5 ^a^ 163.5 ± 39.4 ^b^ 77.6 ± 64.2 ^c^ 40.6 ± 35.6 ^cd^	1.5 ± 0.1 ^d^ 3.6 ± 2.5 ^d^ 5.5 ± 5.2 ^d^ 47.3 ± 48.2 ^cd^ 459.9 ± 56.0 ^a^ 417.5 ± 119.0 ^a^ 252.1 ± 155.0 ^b^ 136.6 ± 109.6 ^c^
DA 2 min 30 s	H_2_O NFL Control abs Control EBR HCS MK TR	1.6 ± 0.2 ^c^ 2.3 ± 0.2 ^c^ 2.3 ± 1.0 ^c^ 1.8 ± 0.5 ^c^ 2.2 ± 0.5 ^c^ 1.9 ± 0.5 ^c^ 16.6 ± 10.2 ^b^ 59.8 ± 30.4 ^a^	1.8 ± 0.2 ^c^ 3.0 ± 0.2 ^c^ 4.1 ± 3.9 ^c^ 2.4 ± 0.6 ^c^ 2.7 ± 0.7 ^c^ 2.4 ± 0.7 ^c^ 80.5 ± 46.7 ^b^ 202.5 ± 93.6 ^a^

**Table 3:** Genes, sequence origins, and designed primers used in the study.

Gene Name	GenBank ID	Forward Primer	Reverse Primer
*COR14*	AY456378.1	GTCAGATTTGGCCGGAAAAC	CTCGGCGTAGATCAACGACTT
*SERK1*	KT281978.1	CGACCACTGCGACCCTAAGAT	CCCTTTCACCCAGTCAAGCA
*SERK2*	KR869962.1	GAGCCTCATCAGCTTGGATCT	GAAGTGTTCTTACAAGGTCACCCC
*actin*	AF111812.1	TCAGTGGTGGTTCGACCATGT	CCGTGATCTCTTTGCTCATACG

## Data Availability

The original contributions presented in the study are included in the article/Supplementary Materials, further inquiries can be directed to the corresponding author/s.

## Supplementary Materials

The following supporting information can be downloaded at: https://www.mdpi.com/article/10.3390/ijms25116010/s1. References [75,76] are cited in the Supplementary Materials.
