# Supplementary material for: Cold Acclimation and Deacclimation of Winter Oilseed Rape, with Special Attention Being Paid to the Role of Brassinosteroids
Source: Int J Mol Sci. 2024 May 30;25(11):6010. doi: 10.3390/ijms25116010 (PMC11172585; doi:10.3390/ijms25116010)
Supplement: Supplementary file 1 [file ijms-25-06010-s001.zip › ijms-3002514-supplementary figures.pdf]

Supplementary-Figures

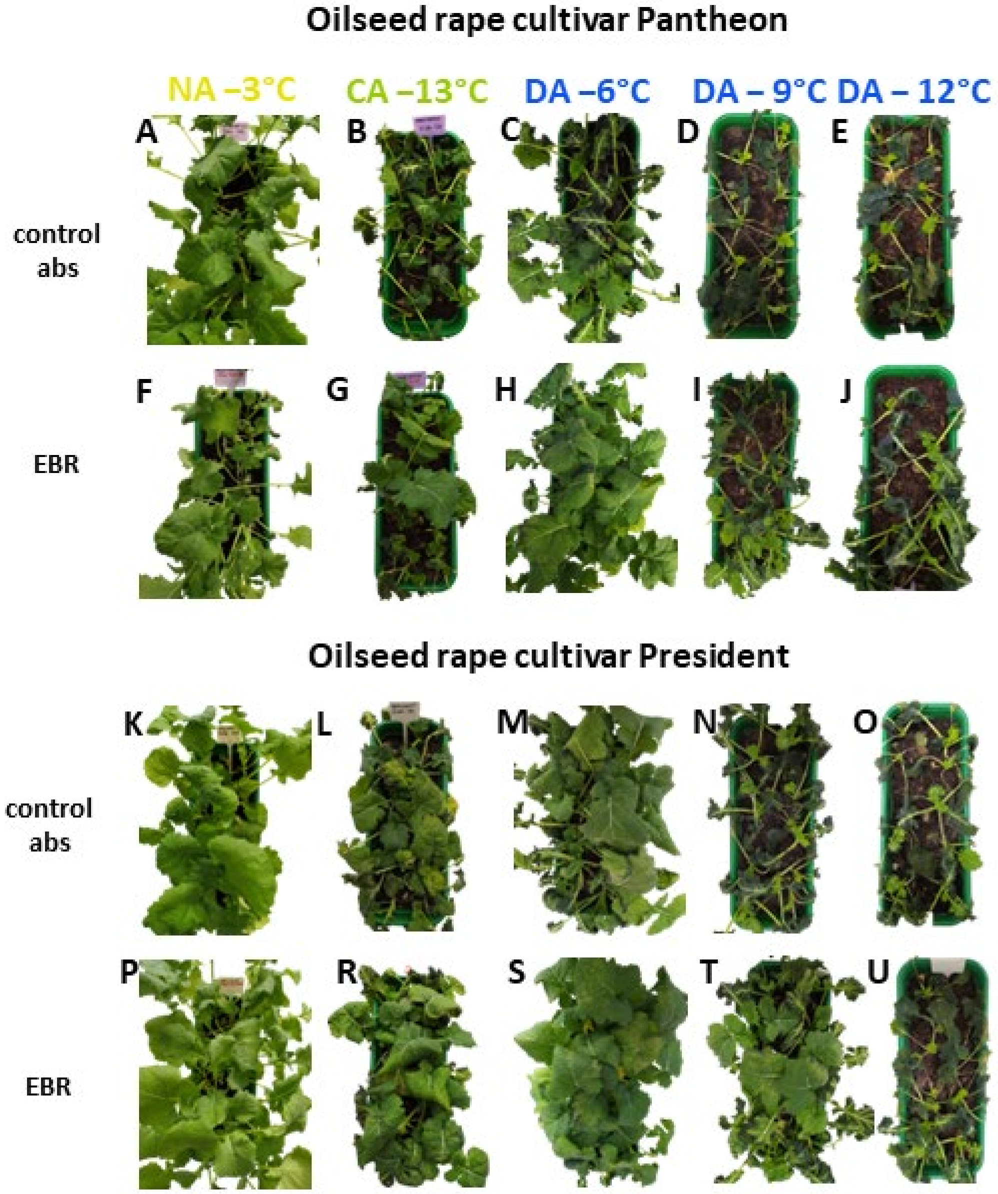

Figure S1. Exemplary photographs of non-acclimated (NA), cold-acclimated (CA), and deacclimated (DA) plants of the oilseed rape cultivars Pantheon and President after the frost tests and a period (two weeks) of regrowth at 12 °C. (A−E) unsprayed Pantheon plants of the absolute control; (F−J) Pantheon plants sprayed with brassinosteroid (EBR—24-epibrassinolide); (K−O) unsprayed President plants of the absolute control; (P−U) President plants sprayed with EBR.

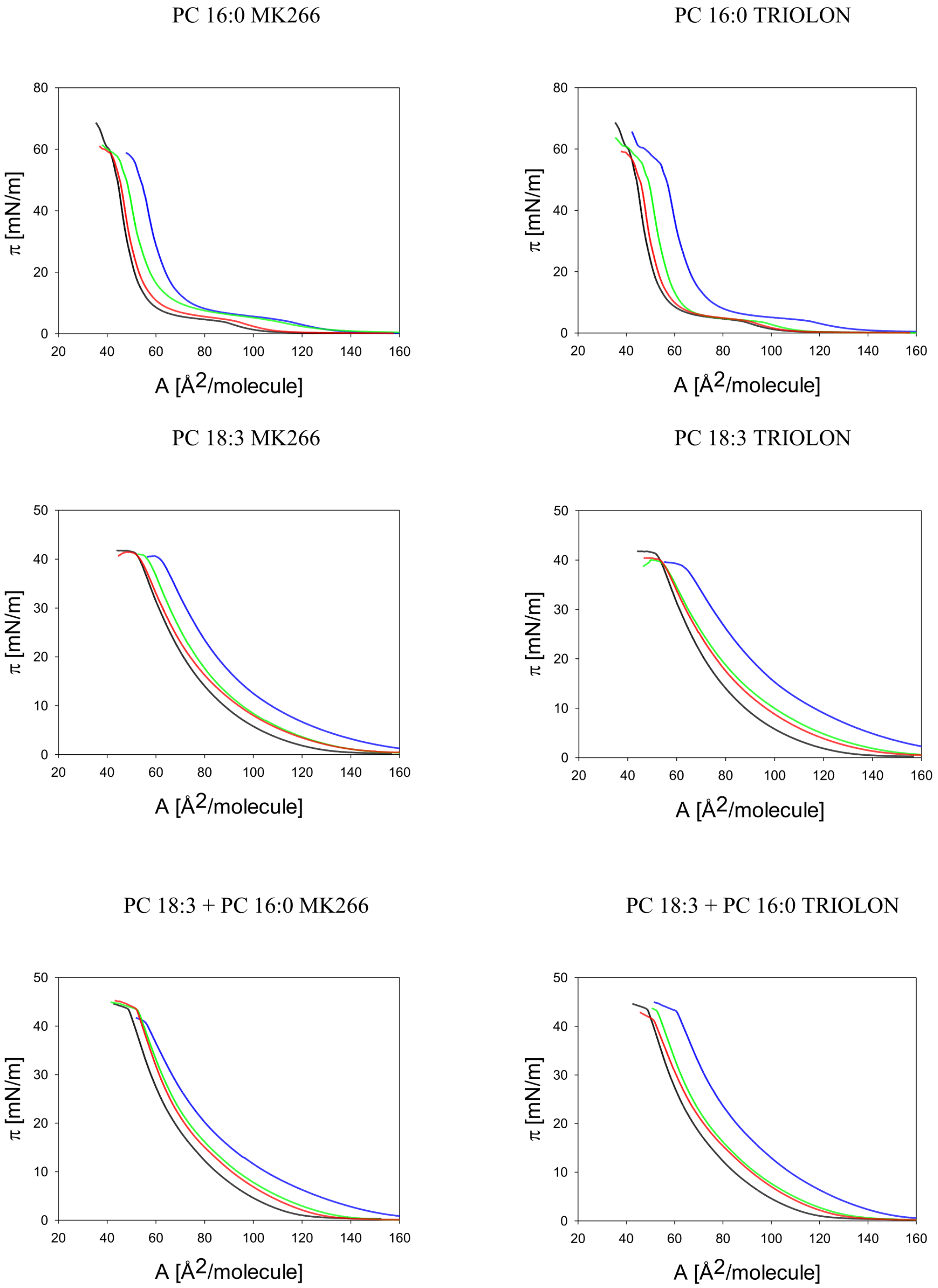

Figure S2. Langmuir isotherms (surface pressure (π) vs. molecular area occupied by lipid) for monolayers of saturated phosphocholine (PC 16:0), unsaturated phosphocholine (PC 18:3), and a lipid mixture (PC 16:0: PC 18:3; 1:1 M:M) without hormones (black line), and with the mixtures of triolon and MK-266 at different molar ratios (lipid–hormone): 16:1 (red line), 8:1 (green line), 4:1 (blue line).

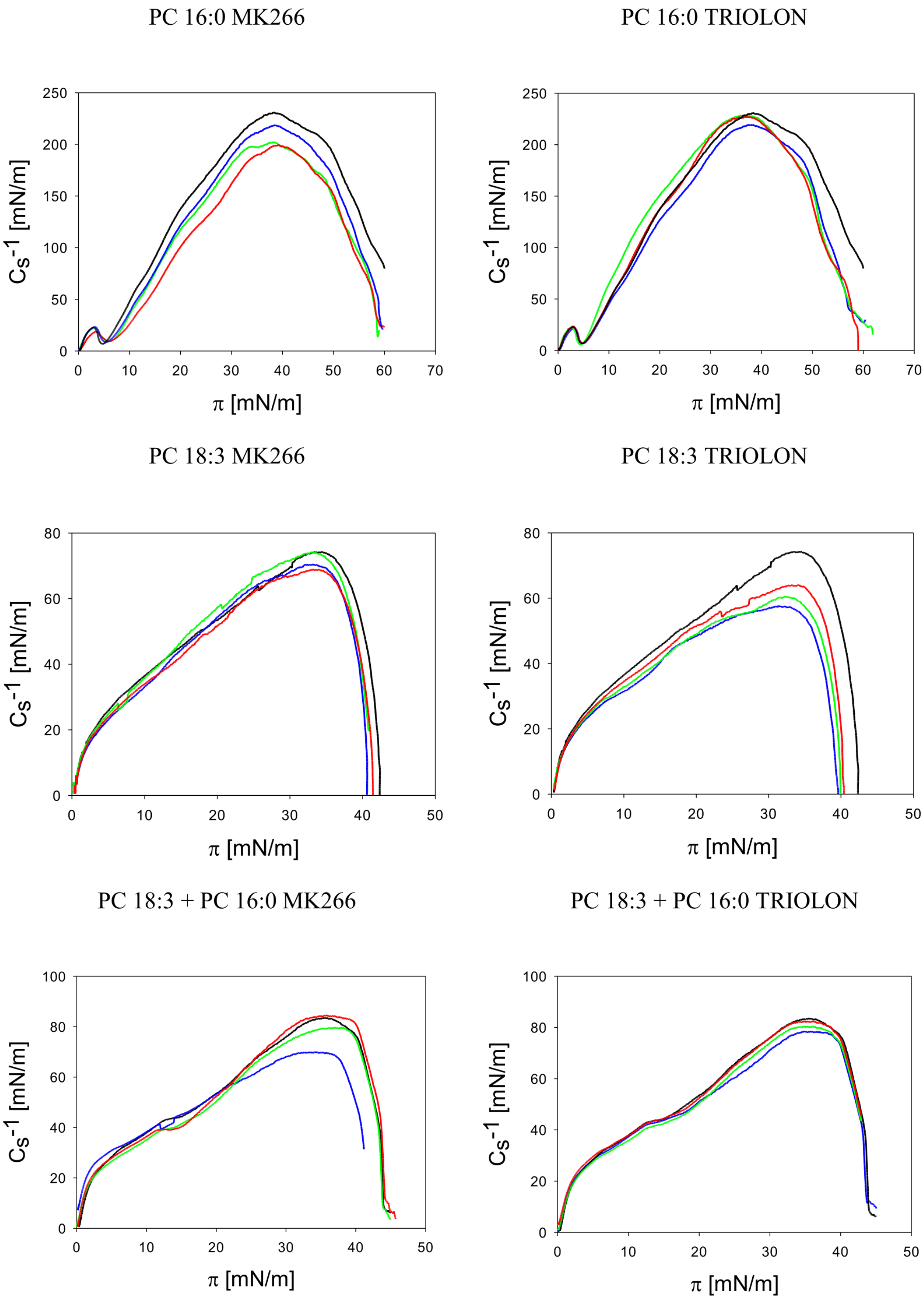

Figure S3. The static compression modulus (C_s^{-1}) as a function of surface pressure (π) for monolayers of saturated phosphocholine (PC 16:0), unsaturated phosphocholine (PC 18:3), and a lipid mixture (PC 16:0: PC 18:3; 1:1 M:M) without hormones (black line), and with the mixtures of triolon and MK-266 at different molar ratios (lipid–hormone): 16:1 (red line), 8:1 (green line), 4:1 (blue line).

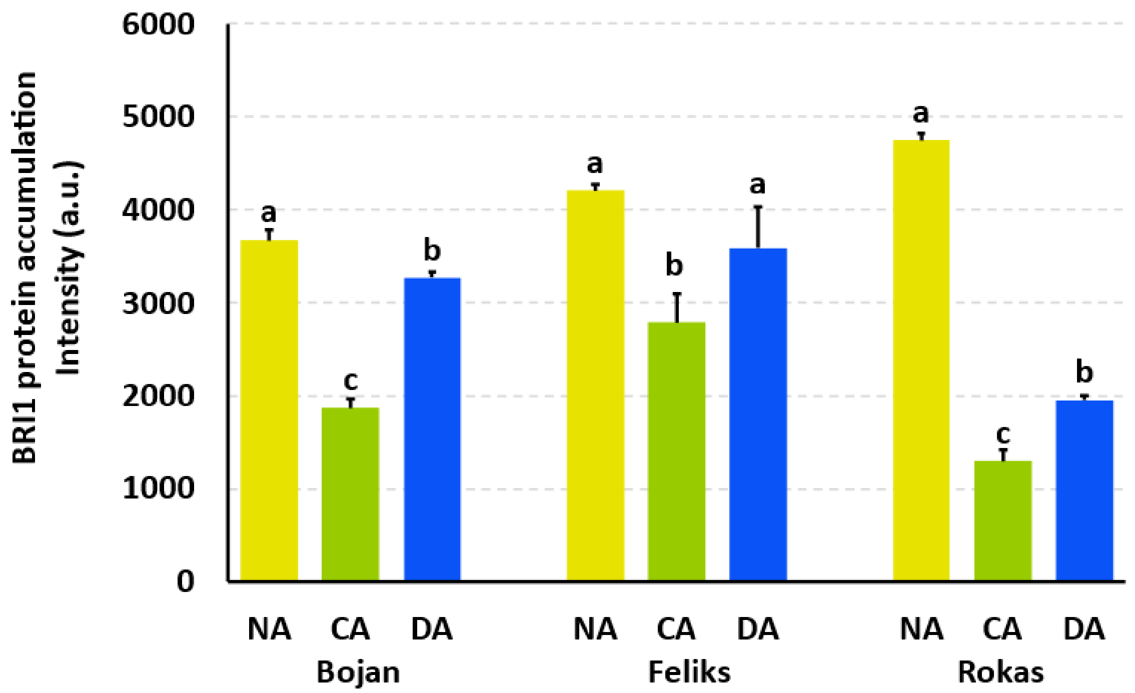

Figure S4. The accumulation of putative BRI1 protein in the leaves of non−acclimated (NA), cold−acclimated (CA) and deacclimated (DA) oilseed rape cultivars Bojan, Feliks and Rokas. The visualised bands corresponding to the level of putative BRI1 protein. 15 µg of protein was loaded onto gel. *Mean values ± SE marked with the same letters do not differ according to Duncan's test ($p<0.05$). Comparison between NA, CA and DA plants within each cultivar.*

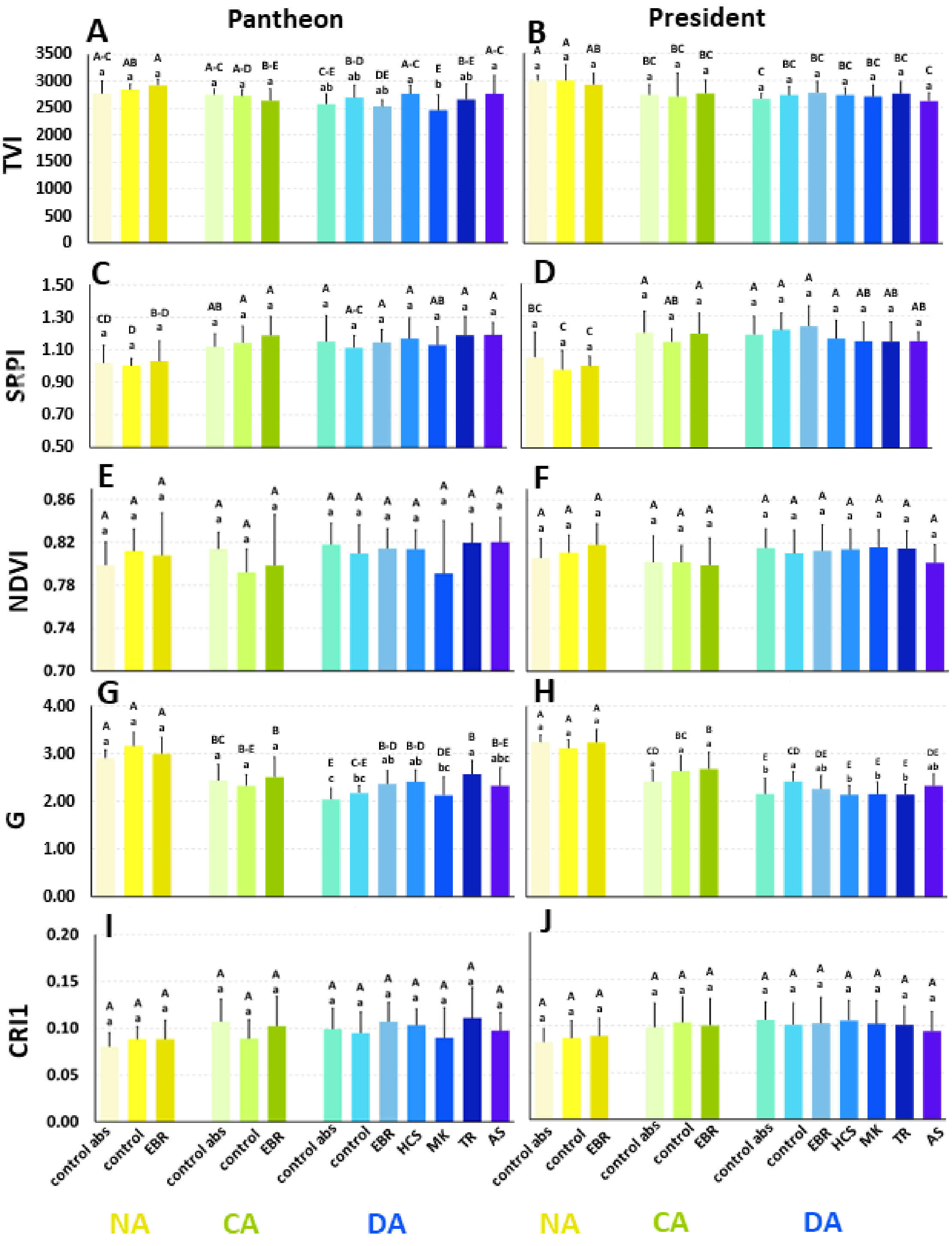

Figure S5. The leaf reflectance parameters of leaves of non-acclimated (NA), cold-acclimated, (CA) and deacclimated (DA) oilseed rape cultivars Pantheon (A,C,E,G,I) and President (B,D,F,H,J). TVI—Triangular Vegetation Index (A,B); SRPI—Simple Ratio Pigment Index (C,D); NDVI—Normalised Difference Vegetation Index (E,F); G—Greenness Index (G,H); CRI1—Carotenoid Reflectance Index 1 (I,J). Control abs—untreated plants; control—plants treated with DMSO (solvent of tested steroids); The other objects represent plants sprayed by brassinosteroids (EBR—24-epibrassinolide; HCS—28-homocastasterone), brassinosteroid analogues (MK—MK-266; TR—triolon), and the regulator Asahi SL (AS). *Mean values marked with the same letters did not differ significantly according to Duncan's test ($p < 0.05$). Lowercase letters—comparisons between treatments within a particular group (NA, CA, and DA plants);*

capital letters—comparisons between treatments of plants of all three groups (NA, CA, and DA plants) together.

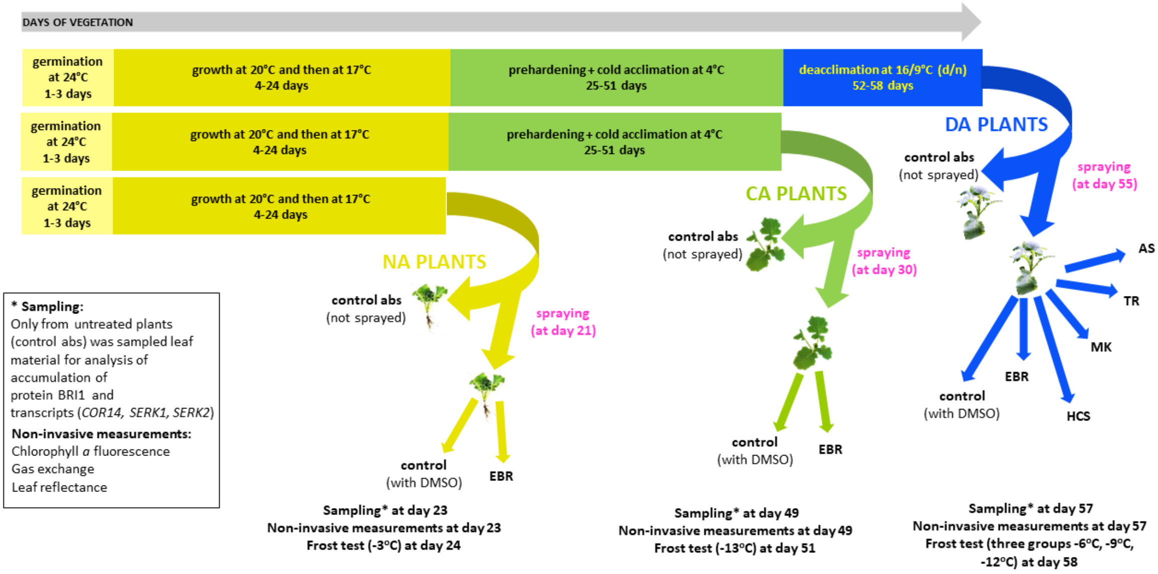

Figure S6. Simplified model of experiment 1. NA plants—non-acclimated plants, CA plants—cold-acclimated plants, DA plants—deacclimated plants; EBR—24-epibrassinolide, HCS—28-homocastasterone, brassinosteroid analogues (MK—MK-266; TR—triolon), AS—regulator Asahi SL, DMSO—solvent of steroids, control abs—absolute control (untreated plants). Non-invasive measurements and frost tests were carried out on all plants. Samples for protein and transcript analysis were collected only from untreated plants of the absolute control (control abs).

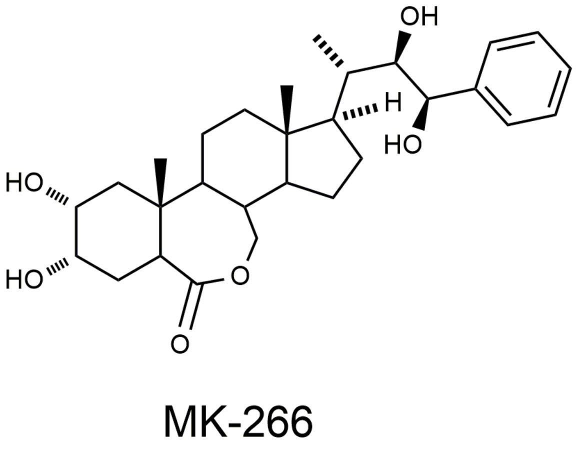

Figure S7. Structure of brassinosteroid analogues MK-266 and triolon. These compounds were synthesised as described in [75,76].
