# Supplementary material for: Cold Acclimation and Deacclimation of Winter Oilseed Rape, with Special Attention Being Paid to the Role of Brassinosteroids
Source: Int J Mol Sci. 2024 May 30;25(11):6010. doi: 10.3390/ijms25116010 (PMC11172585; doi:10.3390/ijms25116010)
Supplement: Supplementary file 1 [file ijms-25-06010-s001.zip › ijms-3002514-supplementary Table S1.pdf]

Table S1. Physicochemical parameters of the Langmuir monolayers: the limiting area per lipid molecule (A_{lim} [Å²]), collapse pressure (π_{coll} [mN/m]) and compression modulus (C_s^{-1} [mN/m]). Statistically significant changes for systems with different molar concentrations (lipid:hormone 4:1 vs. 8:1 vs. 16:1) are noted by uppercase letters for MK−266 and lowercase letters for triolon.

Lipids and BR analogues	A_{lim} [Å^2/molecule]	π_{coll} [mN/m]	C_s^{-1} $_{max}$ [mN/m]
PC 16:0	$57.3^{D/d}$	$59.2±0.1^{A/c}$	$229.3±0.1^{A/a}$
PC 16:0 + MK 4:1	72.0^{A}	$57.0±0.1^{C}$	$218.2±0.1^{B}$
PC 16:0 + MK 8:1	65.4^{B}	$58.3±0.2^{B}$	$202.0±0.1^{C}$
PC 16:0 + MK 16:1	60.0^{C}	$57.1±0.1^{C}$	$199.9±0.1^{D}$
PC 16:0 + TR 4:1	75.2^{a}	$60.9±0.1^{a}$	$220.0±0.2^{d}$
PC 16:0 + TR 8:1	65.9^{b}	$60.4±0.1^{b}$	$226.5±0.1^{c}$
PC 16:0 + TR 16:1	60.3^{c}	$59.0±0.2^{c}$	$227.0±0.1^{b}$
PC 18:3	$93.2^{D/d}$	$41.8±0.1^{A/a}$	$73.8±0.3^{A/a}$
PC 18:3 + MK 4:1	105.0^{A}	$40.2±0.2^{B}$	$70.5±0.1^{B}$
PC 18:3 + MK 8:1	97.8^{B}	$40.5±0.2^{B}$	$74.0±0.1^{A}$
PC 18:3 + MK 16:1	95.7^{C}	$40.1±0.2^{B}$	$68.8±0.2^{C}$
PC 18:3 + TR 4:1	120.0^{a}	$39.4±0.1^{b}$	$58.1±0.1^{d}$
PC 18:3 + TR 8:1	103.3^{b}	$38.6±0.1^{c}$	$60.9±0.1^{c}$
PC 18:3 + TR 16:1	102.0^{c}	$39.6±0.1^{b}$	$63.2±0.1^{b}$
PC 18:3 + 16:0	$82.8^{D/d}$	$43.6±0.2^{A/a}$	$83.5±0.1^{A/a}$
PC 18:3 +16:0 MK 4:1	104.7^{A}	$40.9±0.1^{B}$	$69.7±0.1^{D}$
PC 18:3 +16:0 MK 8:1	90.7^{B}	$43.2±0.3^{A}$	$79.3±0.2^{C}$
PC 18:3 +16:0 MK 16:1	85.8^{C}	$43.3±0.2^{A}$	$84.4±0.2^{B}$
PC 18:3 +16:0 TR 4:1	104.1^{a}	$42.9±0.2^{b}$	$79.0±0.1^{d}$
PC 18:3 +16:0 TR 8:1	93.9^{b}	$43.1±0.1^{b}$	$80.9±0.2^{b}$

PC 18:3 +16:0 TR 16:1	93.3[c]	41.3±0.1[c]	82.7±0.1[c]
